# Cafeteria assessment for elementary schools (CAFES): development, reliability testing, and predictive validity analysis

**DOI:** 10.1186/s12889-018-6032-2

**Published:** 2018-10-03

**Authors:** Kimberly A. Rollings, Nancy M. Wells

**Affiliations:** 1 School of Architecture, University of Notre Dame, 110 Bond Hall, Notre Dame, IN 46556 USA; 2 Design + Environmental Analysis, College of Human Ecology, Cornell University, 1411 Martha Van Rensselaer Hall, Ithaca, NY 14853 USA

**Keywords:** Elementary school cafeteria, Built environment, Assessment tool, Dietary intake, Fruit and vegetable consumption, Healthy eating, Lunch tray photography, Children

## Abstract

**Background:**

Strategies to reduce childhood obesity and improve nutrition include creating school food environments that promote healthy eating. Despite well-documented health benefits of fruit and vegetable (FV) consumption, many U.S. school-aged children, especially low-income youth, fail to meet national dietary guidelines for FV intake. The Cafeteria Assessment for Elementary Schools (CAFES) was developed to quantify physical attributes of elementary school cafeteria environments associated with students’ selection and consumption of FV. CAFES procedures require observation of the cafeteria environment where preparation, serving, and eating occur; staff interviews; photography; and scoring.

**Methods:**

CAFES development included three phases. First, assessment items were identified via a literature review, expert panel review, and pilot testing. Second, reliability testing included calculating inter-item correlations, internal consistency (Kuder-Richardson-21 coefficients), and inter-rater reliability (percent agreement) based on data collected from 50 elementary schools in low-income communities and 3187 National School Lunch Program participants in four U.S. states. At least 43% of each participating school’s students qualified for free- or reduced-price meals. Third, FV servings and consumption data, obtained from lunch tray photography, and multi-level modeling were used to assess the predictive validity of CAFES.

**Results:**

CAFES’ 198 items (grouped into 108 questions) capture four environmental scales: room (50 points), table/display (133 points), plate (4 points), and food (11 points). Internal consistency (KR-21) was 0.88 (overall), 0.80 (room), 0.72 (table), 0.83 (plate), and 0.58 (food). Room subscales include ambient environment, appearance, windows, layout/visibility, healthy signage, and kitchen/serving area. Table subscales include furniture, availability, display layout/presentation, serving method, and variety. Inter-rater reliability (percent agreement) of the final CAFES tool was 90%. Predictive validity analyses indicated that the total CAFES and four measurement scale scores were significantly associated with percentage consumed of FV served (*p* < .05).

**Conclusions:**

CAFES offers a practical and low-cost measurement tool for school staff, design and public health practitioners, and researchers to identify critical areas for intervention; suggest low- and no-cost intervention strategies; and contribute to guidelines for cafeteria design, food presentation and layout, and operations aimed at promoting healthy eating among elementary school students.

**Electronic supplementary material:**

The online version of this article (10.1186/s12889-018-6032-2) contains supplementary material, which is available to authorized users.

## Background

National strategies to reduce childhood obesity include creating school food environments that promote healthy eating (e.g., [1, 2]). In the U.S., nearly 99% of public schools participate in USDA breakfast and lunch programs that offer free- and reduced-price meals (FRPM), in addition to full-price meals, to students based on financial need [3]. Children consume as many as two meals and snacks per day while at school [3], accounting for 19–50% of their daily caloric intake [4]. Despite well-documented health benefits of fruit and vegetable (FV) consumption [5–7], approximately 80% of U.S. school-aged children, especially low-income youth, fail to meet national dietary guidelines for FV intake [8]. FV - along with milk - consumption is highly correlated with the quality of students’ diets [9, 10]. Several studies found that FV are thrown away more than any other food item during school lunch periods [11, 12]; among school children, 40% of cooked vegetables, 30% of salads, and 20% of fruits were wasted daily [12]. Considering that federally-funded meal programs feed more than 31 million students daily, the school cafeteria environment has great potential to encourage healthy eating.

A growing literature suggests that school-based environmental interventions affect health behaviors, including students’ selection and consumption of healthy foods. In addition to social, cultural, economic, policy, and psychological factors, school cafeteria physical attributes including design, display, and layout at multiple environmental scales can affect meal choices, especially when students are faced with long lines and short meal times [13–15]. Physical environment intervention suggestions to promote healthy eating include updating interior design; reducing crowding; creating attractive serving displays and seating areas; selecting appropriately-sized serving trays, plates, and bowls relative to desired portion sizes; and changing the way individual food items are prepared and presented. For example, attractive, well-lit cafeterias with windows and a layout that provides convenient access to healthy foods can affect eating behaviors [15–17]. Placing fresh fruit by the cafeteria checkout rather than earlier in the serving line is associated with an increase in purchases, as are well-lit fresh fruit displays [13, 18–20]. Manipulating availability of healthy items; rearranging the order and placement of food items in serving lines; providing appropriate display and dining furniture; serving tray availability and design; manipulating portion sizes via bowl and plate sizes; and altering presentation of individual food items, as well as item packaging, all have the potential to affect food selection and consumption [15, 17, 21–34]. Despite the increase in research and design guidelines aimed at promoting healthy eating in school cafeterias, no comprehensive, reliable, or validated assessment tool exists to quantify physical attributes of school cafeterias across environmental scales, from interior design characteristics to individual food items. Quantitative data are needed to develop and prioritize evidence-based interventions and design guidelines for elementary school cafeteria environments that promote healthy eating.

The Cafeteria Assessment for Elementary Schools (CAFES) study had three aims: 1) to identify elementary school cafeteria physical attributes at multiple environmental scales [e.g., room (interior design and ambient environment), table and display (dining table and display areas), plate (lunch tray), and individual food items (e.g., [17]) linked to children’s selection and consumption of healthier foods; 2) to create a comprehensive assessment tool via reliability testing; and 3) to evaluate the predictive validity of the tool. Scores resulting from the developed tool were intended to highlight specific areas on which to focus intervention strategies and inform the development of low- or no-cost interventions that can immediately be implemented. By focusing on elementary schools, USDA-funded National School Lunch Program participants, and free- and reduced-price meal (FRPM) recipients, the CAFES tool would benefit high-risk and underserved FRPM student populations and contribute to younger students’ development of healthy eating habits. The following sections discuss CAFES item selection and development, reliability testing, and predictive validity analysis.

## Methods

The Methods section is organized by the three distinct parts of the CAFES study: CAFES item identification (literature review, expert panel review, and pilot testing; CAFES part 1: Item idenfication), reliability testing (CAFES part 2: Reliability), and predictive validity testing (CAFES part 3: Predictive validity analysis).

### CAFES part 1: Item identification

#### Literature review procedures

Literature based in public and environmental health, environmental psychology, behavioral economics, and socioecological models was reviewed to identify physical environment attributes that promote healthy eating, especially among elementary school-aged students [3, 13, 17, 18, 24, 35, 36]. Literature included empirical studies, literature reviews, USDA reports, and existing environmental assessment tools (e.g., [17, 37–40]). Although most literature focused on school cafeteria settings, relevant studies conducted in residential, food retail, and workplace environments were also included. A wide range of attributes within elementary school cafeteria environments hypothesized to promote selection and consumption of healthier food was identified (e.g., interior design, food presentation techniques), as well as novel features not commonly found in the literature but that may affect selection and consumption of healthier food (e.g., noise, student circulation, leftover food-sharing tables). Most identified features were objectively measureable, but some subjective items were included (e.g., cafeteria design attractiveness).

A 400-item draft assessment tool was created based on attributes of cafeteria environments hypothesized to affect healthy eating identified in the literature review. Item measures required school principal and food service manager interviews and an in-person “walk-through” or observation of the cafeteria areas. CAFES items were grouped into interview and observation items, and by space: kitchen/preparation area, serving area, and dining area.

#### Expert panel review procedures

During CAFES development, face validity was evaluated via feedback from five experts invited to review CAFES items for representativeness and relevance. Experts represented the fields of behavioral economics, nutrition, environmental psychology, human development, health, and design. Prior to reviewing the CAFES draft, each expert received a project description, a CAFES tool draft, a description of CAFES data collection and scoring procedures, and three questions concerning the representativeness and relevance of CAFES items:

- *Do CAFES items represent a range of environmental scales?*
- *Are any key environmental attributes missing from the assessment tool?*
- *Do you have suggestions for improving the data collection and scoring procedures?*

Feedback was provided via phone calls, meetings, and emails, and included clarifications to, modifications to, and additions of specific items as well as training and scoring procedures.

#### Pilot testing procedures

Four researchers were trained to use the CAFES draft protocol by first coding 10 sets of example school cafeteria photographs. Coding discrepancies were discussed, CAFES item text and instructions were modified for clarification, and cafeteria photo evaluations were repeated until agreement was reached on all coding conventions. Once observers reached 90% inter-rater reliability (2 h), measured by percent agreement, they piloted the CAFES tool at two local elementary schools. CAFES observations included interviews with school principals and food service staff; walk-through observations of the cafeteria preparation, serving, and dining areas; and sketching and photographing those three spaces for further coding after completion of on-site interviews and observations. Initial CAFES observations required 45–120 min to complete at each school, depending on interview duration and whether students were present in the eating and serving areas.

### CAFES part 2: Reliability testing

#### Participants

CAFES reliability testing was based on a cross-sectional sample of 50 elementary schools (3187 students, total) in New York (*n* = 16), Iowa (*n* = 17), Arkansas (*n* = 10), and Washington (*n* = 7) participating in the Healthy Gardens, Healthy Youth (HGHY) pilot program. The 2.5-year, USDA-funded, randomized school garden pilot project included examination of FV consumption in elementary schools (Wells, N.M., lead researcher). Cooperative Extension educators recruited schools from low-income rural, urban, and suburban communities; without a school garden; and with at least 50% of students qualifying for FRPM at the time of selection [41]. Trained researchers in New York and Washington and trained Cooperative Extension Educators in Iowa and Arkansas collected CAFES data. The CAFES study was deemed exempt by the Cornell University and University of Notre Dame Institutional Review Boards.

#### Procedures

CAFES observations were repeated at participating schools using the Part 1 CAFES version containing hundreds of items. To determine which CAFES items to retain or eliminate, identify measurement scales and subscales, and assess the reliability of the resulting CAFES tool, measures of internal consistency, inter-item correlations, and inter-rater reliability were calculated. First, each CAFES item was dichotomously coded into negative (0 = barrier to healthy eating) and positive (1 = facilitator of healthy eating) point values using IBM SPSS Statistics for Windows (IBM Corp., Version 23.0). Per Part 1 expert panel review feedback, binary item coding facilitated scoring and reliability testing.

The large number of CAFES items and modest school sample size precluded use of factor analysis to reduce the number of items. Therefore, item variability and inter-item correlations were calculated and served as criteria for item omission [42]. CAFES items were grouped according to each of the four environmental scales and themes (subscales) identified in the Part 1 literature review. Then, items with the lowest variability (i.e., an individual item with little to no variation across schools) and items with low inter-item correlations were omitted. Each time an item was omitted, Kuder-Richardson 21 (KR-21) coefficients, a measure of internal consistency for binary items [43], were calculated. The procedure was repeated until KR-21 coefficients of at least .70 and acceptable average inter-item correlations were achieved for the overall CAFES tool, four measurement scales, and emergent subscales [42]. Schools lacking at least 50% of items within any measurement scale or subscale were excluded from analysis of that scale or subscale (see Additional file 1: Tables S1-S3, for school sample sizes – ranging from 20 to 36 schools – applicable to each CAFES scale and subscale).

CAFES scores (percentage out of 100%) were then calculated by summing all points and dividing by the total number of points. Scoring calculations were repeated for each CAFES measurement scale and subscale. Scores indicated how well cafeteria environments promoted or inhibited FV selection and consumption overall, and within each scale and subscale. Several CAFES items were also designated as possible “not applicable” items. For example, a school without a kitchen was awarded zero points once, but all subsequent kitchen items were deemed not applicable and associated points were deducted from the total points possible. Inter-rater reliability of the revised CAFES tool was assessed by calculating the percent agreement among at least three of four trained researchers’ CAFES responses at four additional elementary schools in a fifth state, not part of initial data collection.

### CAFES part 3: Predictive validity analysis

#### Participants

Of the 50 schools that participated in reliability testing, 44 provided FV servings and consumption data via lunch tray photography (2506 students). Students who brought lunches from home (519 meals, 216 students); 82 students with missing, dark, or blurry photographs; and schools missing at least 50% of any CAFES scale or subscale items were eliminated from predictive validity analysis [44]. Two predictive validity analysis subsamples remained: 29 schools (1544 students) supplied complete CAFES items and 16 schools (1069 students) supplied complete items for the four CAFES measurement scales. Subsample demographics are displayed in Additional file 1: Table S4. Additional file 1: Tables S5a-c display FV outcome summary statistics for the 44 schools that collected lunch tray photography data, and the two predictive validity testing subsamples.

#### Constructs and measures

At the school-level, CAFES observation data, student population, percentage of students eligible for FRPM, percentage of minority students, and urbanity were obtained from the HGHY study. Urbanity, or whether a school was in an urban, rural, or suburban location, was determined based on U.S. census definitions of population density [45]. Individual student gender, grade level, FRPM eligibility, ethnicity, age, and body mass index (BMI) were reported by parents in a survey distributed as part of the HGHY study.

**Fig. 1:**
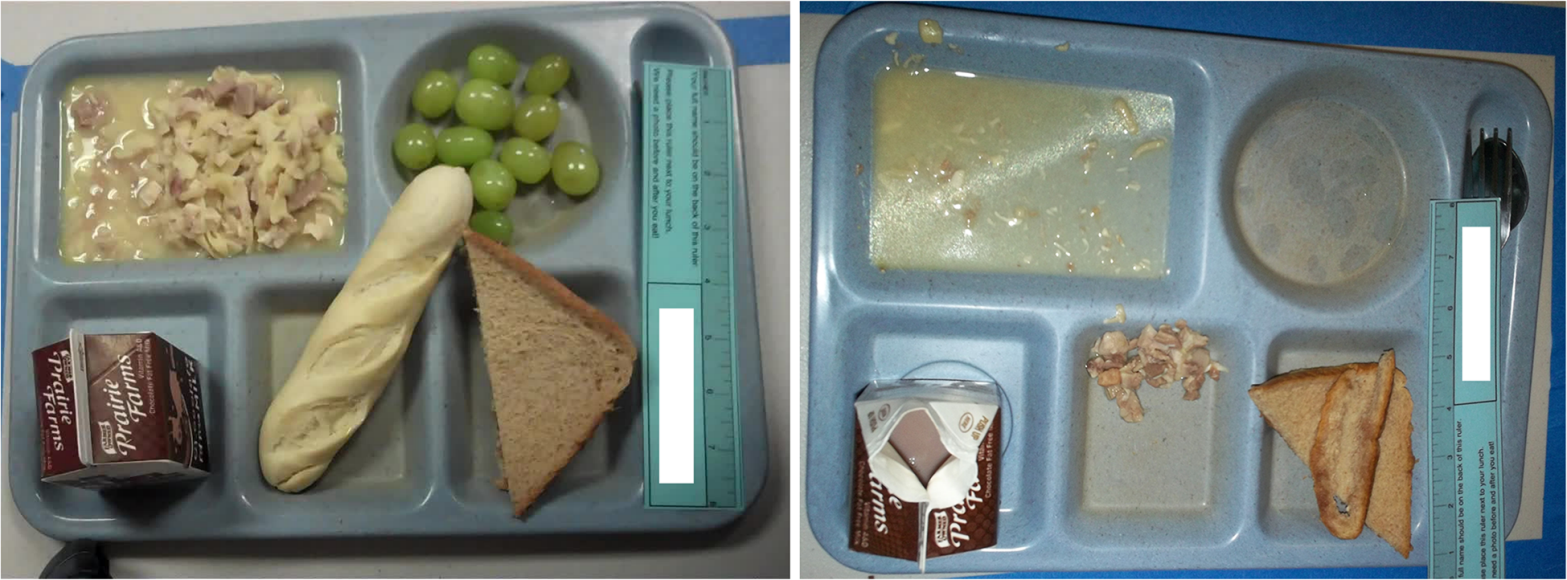
Lunch Tray Photograph Pairs. Two examples of “pre” (left) and “post” (right) lunch tray photography pairs

At the individual student level, FV servings and consumption outcome data were obtained by attaching laminated identification number cards to student lunch trays and photographing trays twice: once immediately after students were served, and again after they ate [44, 46]. Digital Food Image Analysis (DFIA) software analyzed “before and after” lunch tray photograph pairs (Fig. 1) using school menus, cafeteria production records, and the USDA’s nutrient database. DFIA validity was previously assessed via comparisons to dietitians’ digital observations [44]. FV servings and percent consumption recorded by both methods were moderately and strongly correlated, respectively. Correlations were either comparable to or more robust than prior studies assessing dietary assessment method validity [44]. DFIA analyses yielded four quantities used to calculate FV outcomes for the CAFES study: fruit served, fruit consumed, vegetables served, and vegetables consumed, all measured in grams.

Predictive validity testing included both FV serving and consumption outcomes. Distinguishing between foods available to students, foods students choose or are served, and foods students actually consume is important because factors affecting selection and consumption differ [47, 48]. Although selection and consumption of fruits verses vegetables may also differ, only combined FV measures were analyzed. Combined FV measures addressed within- and between-school variations in the number of FV options available to students, as well as the number of allowable FV servings. For example, students could select two fruits and one vegetable at one school, but one fruit and two vegetables at another. Therefore, FV servings and consumption data were averaged from lunches on three separate days to yield two outcome variables: FV served and FV consumed. Percentage consumed of FV served (percent consumed) was then calculated by dividing FV consumed by FV served, and allowed for comparisons among FV items with standard serving sizes that varied between schools [44].

Furthermore, per expert panelist feedback, combined FV serving and consumption measures focused on FV “side items,” rather than both FV entrees (e.g., tomato sauce) and sides (e.g., whole fruit, applesauce, steamed vegetables, etc.). Finally, predictive validity testing examined foods, not beverages, for two reasons. First, beverage consumption from opaque milk containers could not be documented via photographs for DFIA analysis. Second, all students were served one prepackaged carton or bottle of low-fat milk. This packaging type creates a “natural consumption unit” [17] that can lead diners to consume the entire unit, also known as unit bias [49]. Although future work could examine associations between CAFES scores and student milk selections (e.g., flavored or unflavored), CAFES predictive validity testing excluded beverages due to the lack of consumption data and variability in servings.

#### Procedures

Predictive validity was assessed using Hierarchical Linear Modeling software (Version 7.0; [50]) to determine whether (A) CAFES total and (B) four measurement scale scores significantly predicted FV servings and consumption outcomes (see *CAFES part 2: Reliability testing* results for measurement scales and subscales). The two-level data structure consisted of student level controls (grade, gender, and BMI; age was excluded due to missing data and high correlation with grade) nested within school level CAFES scores (A-CAFES total and B-four scale scores) and school level controls (percent of students receiving FRPM, percent minority student population, urbanity). The sample size did not permit exploring a three-level model (students within classes within schools). All variables, except for CAFES scores, were grand-mean centered. Two sets of multilevel models containing the following school-level predictors were run: A) CAFES total score and B) four CAFES scale scores. FV outcome variables included FV served and FV percentage consumed.

## Results

### CAFES part 1: Item identification

#### Literature review

Table 1 displays themes and four environmental scales drawn from the literature review that guided preliminary CAFES item selection. Numerous environmental attributes were included in the initial CAFES version so that the resulting tool could be used to assess widely varying elementary school cafeteria environments. “Room scale” physical attributes, related to the interior design of kitchen, serving, and dining areas, that potentially affect healthy eating included ambient environment, appearance, layout, and advertising. Table/display scale attributes described the appearance of furnishings, equipment, and surfaces from which foods and beverages are served and consumed [17]. Items included size, shape, surface material, and condition of tables, counters, and serving displays, as well as availability, display and layout, serving method, and variety of items served within serving and dining areas. Plate scale items included the size, shape, transparency, color, and material of lunch trays, plates, bowls, glasses, containers, and utensils [17]. Food scale items described the appearance (e.g., size, shape, texture, color) of individual food and beverage items [17, 51].

**Table 1:** School cafeteria environment assessment themes and example items

Theme	Assessment item examples resulting from the literature review	Environmental Scale [17]
*Availability*	Available food preparation and storage space	Room
	Availability & variety of healthier foods (FV, milk)	Table/display
	Competitive food, beverage, and vending availability	Table/display
	Packaging of food items	Food
*Accessibility: layout, display, visibility, and convenience*	Floor plan layout/circulation	Room
	Food and beverage arrangement and display	Table/display
	Lunch tray use	Plate
	Food preparation (e.g., whole or sliced fresh fruit)	Food
*Naming and labeling*	Creativity of food item naming on menus	Table/display
	Labeling of individual food items	Table/display
*Advertising/signage*	Healthy eating promotion / unhealthy item advertising	Room
*Ambient environment*	Temperature, odor	Room
	Crowding and noise	Room
	Lighting: natural and artificial	Room
	Appearance/structural condition and quality	Room
	Clutter, cleanliness, and maintenance	Room
	Seating arrangement and furniture	Table/display

#### Expert panel review

Expert panel review feedback ranged from suggested improvements to training protocols, observation procedures, and CAFES instructions to item adjustments and scoring. One panelist noted, based on prior work, that CAFES observations should not be completed when pizza is served as a meal item because students are likely to select and consume that favorite item more than others, regardless of environmental influences. This panelist also encouraged focus on side dishes rather than entrees, as most fruit and vegetable content of school meals is found in those dishes. Another panelist noted that some policies should be documented during CAFES observations as they have been found to affect eating behaviors (e.g., available time for lunch, whether recess occurs before or after lunch, and whether meals are prepared on- or off-site). Improvements were also suggested to CAFES items relating to general serving methods and the display and serving of milk. Scoring suggestions included dichotomizing results to facilitate calculations, which was implemented in CAFES Part 2. The CAFES tool and procedures were modified per the panel experts’ recommendations. Policy items unrelated to the physical environment, however, were not added to CAFES [37].

#### Pilot testing

**Fig. 2:**
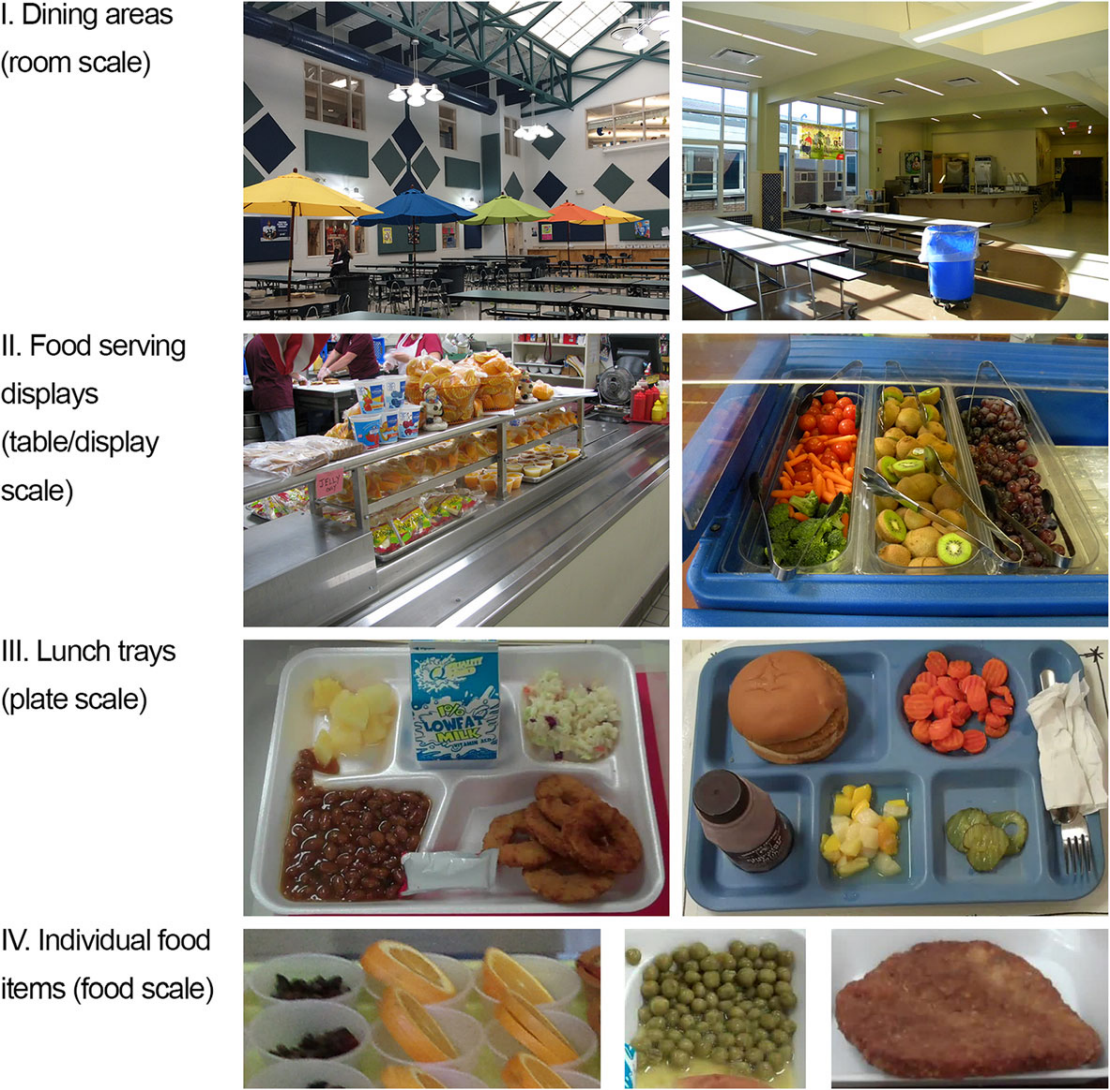
Example CAFES Photographs. Example CAFES photographs from school cafeteria dining areas (row I), serving displays (row II), serving trays (row III), and individual food items (row IV)

Example photographs from pilot CAFES observations are displayed in Fig. 2. Based on pilot testing, CAFES item order and procedures were revised for efficiency and to indicate whether items should be completed with or without students present. For example, measuring occupied dining areas was difficult and drew attention to observers, so revised procedures suggest those items be completed without students present. Pilot testing also revealed discrepancies between interview and observation data. Additional exploration revealed that food service staff needed to be reassured by both the Principal and CAFES observers that the environment – not the staff – was being evaluated during CAFES observations. Staff were then comfortable providing complete and accurate responses that did not conflict with observations.

Additionally, item coding was revised. For example, the serving tray area (size) variable was recoded. Smaller trays were originally coded positively based on studies that found an association between larger plate and bowl sizes and increased intake among adults [17, 52]. CAFES observations and interviews, however, indicated that smaller and less-sturdy serving trays (e.g., foam or thin, disposable plastic) were difficult for students to handle and may lead to decreased FV servings when students serve themselves**,** and lower FV consumption. Larger, sturdier reusable plastic trays were observed to be more appropriate for elementary school students to carry and balance while obtaining food. Results and the final CAFES tool, therefore, negatively code smaller tray sizes with a “0” and not a “1.”

### CAFES part 2: Reliability testing

Table 2 describes the schools and students that participated in CAFES reliability testing. Schools were primarily in urban and rural locations with an average of 391 students, 69% FRPM recipients, and 53% minority students. Missing student level data was especially challenging to obtain, as indicated by missing data.

**Table 2:** Descriptive Statistics of 50 CAFES Schools

School Level Variable	Variable Levels	*N*	Total		Student Level Variable	Variable Levels	*n* /3187	Total	
			#	%				#	%
Urbanity	Urban Rural Suburban	50	20 22 8	40 44 16	Gender	Male Female *Missing*	2022	930 1092 *1165*	29 34 *37*
Location	Arkansas Iowa New York Washington	50	10 17 16 7	20 34 32 14	FRPM recipients	Full Reduced Free *Missing*	2007	662 198 1147 *1180*	21 6 36 37
					Grade level	2nd 4th/5th *Missing*	2506	1190 1316 *681*	38 41 *21*
Student population *(# students)*	Mean SD Range	50	391 168 120–894		Ethnicity	White Black Hispanic Asian Native American Other *Missing*	2033	1068 418 309 58 28 152 *1154*	33.5 13.1 9.7 1.8 0.9 4.8 *36.2*
% FRPM recipients	Mean SD Range	50	69% 18% 43–100%		BMI	Mean SD Range *Missing*	982	19.5 5.0 10.2–46.4 *2205*	
Ethnicity *(% minority students)*	Mean SD Range	50	53% 32% 1–100%		Age (years)	Mean SD Range *Missing*	2060	8.4 1.2 6–12 *1127*	

*FRPM* Free- and reduced-price meal

*BMI* Body mass index

Brief descriptions of the final 198 CAFES items (grouped into 108 questions) relevant to FV selection and consumption based on reliability testing are provided in Table 3. Table 3 identifies the four CAFES measurement scales that address four environmental levels (room, table/display, plate, and food), six room subscales (ambient environment, appearance, window characteristics, layout and visibility, signage promoting healthy eating and physical activity, and kitchen and serving area-specific attributes), and five table/display subscales (eating area furniture; meal item availability; meal item display, layout, and presentation; serving method; and meal item variety) that resulted from reliability testing. No reliable plate or food subscales emerged based on testing. Example excluded items that did not meet selection criteria and items beyond the scope of CAFES are also noted. Additional files 2 and 3 contain the final CAFES tool and scoring procedures.

**Table 3:** Four CAFES neasurement scales, room and table/display subscales, and individual CAFES item descriptions

**ROOM SCALE** (50 total points)^a^ *Excluded item examples: cafeteria color, material, decoration (subjectivity); kitchen/serving area size (lack of variability).*					
Ambient Env. (9)	Appearance (9)	Windows (8)	Layout & Visibility (8)	Healthy Signage (2)	Kitchen & Serving Area (14)
Eating area temperature (2), odor, crowding (2), ceiling height, lighting, noise, music	Eating area attractiveness, physical condition, furniture condition, clutter, cleanliness; serving area attractiveness, physical condition, clutter, cleanliness	Eating area window presence, condition, quantity, view of nature, operability, transparency; window screen presence; window treatment presence	Student circulation, plan obstructions, menu location, lack of display space, lack of prep area, food/beverage visibility from cafeteria, vending machine visibility from eating area	Presence of healthy & unhealthy diet or physical activity promotional signage (2)	Lunch prepped at school/not; serving area equipment condition, lighting; kitchen presence, attractiveness, cleanliness, clutter, lighting, physical condition, equipment condition & availability, window presence, storage space availability (2)
**TABLE/DISPLAY SCALE** (133 total points) *Excluded item examples: serving item order, serving vessel fullness/size, spot lighting, and serving surface color/material (lack of variability);* *Beyond CAFES scope: Kitchen and serving area equipment inventory.*					
Furniture (4)	Availability (77)	Display Layout/Presentation (14)		Serving Method (19)	Variety (19)
Eating area furniture attractiveness, table shape; seating (bench or individual seats; attached or moveable)	Weekly availability: food items (55), a la carte items (6), beverage items (10); fundraisers (2), vending availability (2); age appropriate portion sizes; ice cream cooler availability	Fruit presentation (1), FV close to register (1), FV in first 3 visible items (1), milk layout (2), menu item naming (1), food item labeling (1), serving area food attractiveness (1), milk location (4), ice cream lid transparency (1), out of reach/by request only items (1)		Tray rest available, serving tray use, self-serve option & for which items (4); large trays or premeasured portions (3), packaging transparency (3); sharing table availability, second servings allowed (2), offer vs. serve (4)	Weekly availability: more than one main course (6), fruit (6), vegetable (6) offered; milk quantities offered
**PLATE SCALE** (4 total points) *Excluded item examples: serving tray color, number and size of serving tray compartments, packaging of items on tray (high variability).* *Beyond CAFES scope: serving utensil size/color, food/beverage packaging characteristics of items on serving trays.*					
Serving tray area (1), choice of color (1), and material (Styrofoam/weak plastic containers or not; 1); utensils (forks, knives, & spoons available or not; 1)					
**FOOD SCALE** (11 total points) *Excluded item examples: individual food item packaging, labeling, and presentation.* *Beyond CAFES scope: food color, temperature, taste, texture, attractiveness, food preferences.*					
Reheat frequency (6), avg # fruits/meal (1), avg # vegetables/meal (1), # meals w/ breaded/fried item (1); % raw FV (1); fresh fruit whole or sliced (1)					

^a^Parenthetical numbers indicate the total points from internally consistent CAFES items based on reliability testing

*FV* Fruits and vegetables

Table 4 displays CAFES scores (total, four measurement scales, and subscales), descriptive statistics, and internal consistency results (KR-21 coefficients). KR-21 coefficients exceeded the 0.70 threshold for the total CAFES score (0.88) and the room, table/display, and plate scales (> 0.70). The 51% mean total CAFES score (range of 35–64%, out of 100%) indicated that CAFES schools could benefit from additional environmental supports of healthy eating behaviors. Few studies have examined the relationship between room scale items in school cafeteria settings and healthy eating outcomes among children. CAFES schools scored highest, on average, at the room scale. Because changing room scale attributes such as ventilation systems, floor plans, and natural and artificial lighting can be expensive, room scale scores suggest that CAFES schools might benefit from less expensive interventions at other environmental scales. Averaging only 43%, CAFES schools would benefit most from table/display scale interventions.

**Table 4:** CAFES scores, descriptive statistics, and reliability analyses

CAFES score *Subscale score*	n^a^	# items^b^		CAFES Score (out of 100%)				CAFES Score & Reliability Analyses					
				Mean^c^	SD	Range		Skewness^d^ (SE)		Kurtosis^e^ (SE)		KR-21^f^	Mean r^g^
ROOM SCALE	38	46	*(50)*	70.10%	10.13%	43.90% -	87.50%	− 0.296	(0.383)	−0.384	(0.750)	**0.80**	0.18
*Ambient subscale*	28	7	*(9)*	61.84%	19.82%	28.57% -	100.00%	−0.082	(0.441)	−0.414	(0.858)	**0.75**	0.22
*Appearance subscale*	37	8	*(9)*	75.98%	23.36%	12.50% -	100.00%	−0.908	(0.388^)	0.153	(0.759)	**0.71**	0.23
*Windows subscale*	35	8	*(8)*	53.48%	31.71%	0.00% -	100.00%	−0.408	(0.398)	−1.171	(0.778)	**0.81**	0.44
*Layout subscale*	37	8	*(8)*	91.29%	16.98%	37.50% -	100.00%	−2.190	(0.388^)	4.208	(0.759^)	**0.83**	0.34
*Healthy signage subscale*	37	1	*(6)*	86.47%	34.66%	0.00% -	100.00%	−2.226	(0.388^)	3.120	(0.759^)	n/a^h^	n/a
*Kitchen/Serve subscale*	40	14	*(14)*	63.71%	14.67%	25.00% -	85.71%	−0.563	(0.374)	0.274	(0.733)	**0.71**	0.16
TABLE/DISPLAY scale	36	95	*(133)*	42.64%	6.78%	29.58% -	62.29%	1.014	(0.393^)	1.668	(0.76^)	**0.72**	0.19
*Furniture subscale*	36	4	*(4)*	33.10%	25.70%	0.00% -	75.00%	0.207	(0.393)	−1.079	(0.768)	0.52	0.20
*Availability subscale*	36	56	*(77)*	40.48%	8.17%	25.93% -	62.50%	0.491	(0.393)	0.236	(0.768)	**0.71**	0.17
*Display subscale*	35	8	*(10)*	39.90%	22.95%	0.00% -	85.71%	0.106	(0.398)	−0.794	(0.778)	**0.80**	0.23
*Serving method subscale*	34	11	*(19)*	64.90%	13.47%	36.36% -	90.91%	0.023	(0.403)	−0.564	(0.788)	0.64	0.24
*Variety subscale*	36	16	*(19)*	40.09%	20.42%	18.75% -	93.33%	0.601	(0.393)	−0.522	(0.768)	**0.82**	0.40
PLATE SCALE	37	3	*(4)*	51.35%	44.16%	0.00% -	100.00%	−0.054	(0.388)	−1.804	(0.759^)	**0.83**	0.66
FOOD SCALE	27	5	*(11)*	51.73%	20.94%	20.00% -	100.00%	0.082	(0.448)	−0.441	(0.872)	0.58	0.24
CAFES TOTAL SCORE	36	149	*(198)*	50.54%	5.96%	34.57% -	64.34%	−0.172	(0.393)	.575	(0.768)	**0.88**	0.18

^a^ *School sample size “n” indicates the number of schools that reported at least 50% of CAFES items at the specified scale/subscale*

^b^ *Number of applicable CAFES items (out of total possible CAFES items) with sufficient variability for reliability testing*

^*c*^ *CAFES scores are out of a possible 100%. Each school’s total score was divided by the total # of relevant CAFES items*

^d^ *A measure of data distribution symmetry. A “^” in the SE column indicates skewed data (not between -SE × 2 and + SE × 2)*

^*e*^ *measure of how peaked (+) or flat (−) the data distribution is relative to a normal distribution. A “^” in the SE column indicates a non-normal distribution (not between –SE × 2 and + SE × 2)*

^f^*Bolded text indicates that the measurement scale/subscale met internal consistency criteria (KR-21* *>* *0.70)*

^*g*^ *Mean inter-item correlation*

^h^ *Healthy signage data lacked variability for reliability testing of this subscale. Thus, the final CAFES instrument retained six items concerning healthy signage content, quantity, and location based on available literature*

The food scale did not reach the .70 KR-21 threshold and was only moderately reliable (0.58), likely due to the exclusion of student-level moderators such as food quality perceptions and preferences (see *Discussion*). Other assessment tools focusing specifically on the food and beverage environment that capture these items (e.g., [53]) are needed when targeting improvements to individual food items. Subscale reliability analyses also revealed that the healthy signage (room scale), furniture (table/display scale), and serving method (table/display scale) subscales did not meet the 0.70 KR-21 criterion, likely due to a lack of variability between observed schools for these items. For example, CAFES cafeterias used a few types of standard cafeteria tables and seating that facilitated quick set-up, removal, and cleaning. CAFES schools could, however, be compared to other schools that offer more home-like or alternative furniture options. The subscales were retained in the final CAFES version due to prior research suggesting associations between these items and eating behaviors.

With the exception of the plate scale, mean inter-item correlations within the other three CAFES measurement scales and subscales were low. Low or insignificant Pearson correlations indicated that items within each scale and subscale were, in fact, measuring separate constructs. Inter-item correlation matrices are presented in Additional file 1: Tables S1-S3. Inter-rater reliability of the final CAFES tool, determined using percent agreement, was 90%.

### CAFES part 3: Predictive validity analysis

Predictive validity analyses examined whether CAFES scores were associated with FV servings and consumption data. Overall, students served and consumed more fruit than vegetables. Unlike college students found to consume, on average, 92% of foods they serve themselves [52, 54], elementary school students in this study only consumed, on average, 52–65% of the FV served (Additional file 1: Tables S5a-c). Students in the two predictive validity analyses subsamples (29 and 16 schools) served and consumed higher amounts of FV when compared to all schools that provided lunch tray photography data (44 schools; Additional file 1: Tables S5a-c).

The amount of variance explained by CAFES scores, an indicator of CAFES effect size, was calculated for all predictive validity models. Fully unconditional and partially conditional model results are displayed in Additional file 1: Tables S6a-b, S7a-b, and S8a-b. Fully unconditional model results indicated significant differences in FV serving and percent consumed (*p* < 0.05 for all γ_00_ intercept coefficients), and that there was still unexplained variance in all outcomes at the school level (*p* < 0.05 for all school level μ_0j_ variance components). Partially conditional models including control variables also contained significant unexplained variance. Urbanity and student population were excluded from final models as neither were significant. Missing student level gender and BMI data precluded inclusion of these variables in analyses, resulting in models that accounted for little to no within-student variance, but school-level variance components were significant for all models.

#### Total CAFES

Total CAFES scores significantly predicted FV percentage consumed, but not FV served. A one percentage point increase in total CAFES score was significantly associated with an average 0.92% - or 1.62 g (50 g is approximately one FV serving [55]) - increase in FV percentage consumed (*p* < 0.05), when controlling for grade level, percent FRPM, and percent minority (Table 5). Total CAFES score accounted for 13% of the between-school variance in FV percentage consumed (Additional file 1: Table S9), likely due to the relatively limited variability among CAFES items within in this sample. FV serving outcomes were not significantly predicted by total CAFES scores because serving-specific outcomes are likely associated with serving area-specific CAFES items.

**Table 5:** Predictive validity: fully conditional model with total CAFES score

	% FV CONSUMED^a^		Final estimation of fixed effects^a^				
Level	Fixed Effect	*n* ^b^	*Coefficient*	*SE*	*t-ratio*	*d.f.*	*p-value* ^c^
*For Intercept, β*_*0*_	γ_00_ Intercept	*29*	0.18	0.22	0.83	25	0.416
	γ_01_% FRPM		−0.12	0.21	−0.60	25	0.554
	γ_02_% Minority		−0.03	0.10	−0.33	25	0.744
	γ_03_ CAFES score		0.92	0.42	2.17	25	**0.040**
*For Grade, β*_*1*_	γ_10_ Intercept	*1441*	0.01	0.03	0.25	1514	0.806
			Final estimation of variance components				
	Random Effect		*Variance component*	*SD*	*Χ* ^*2*^	*d.f.*	*p-value* ^c^
	Level 2 *μ*_0j_		0.015	0.122	227.70	25	**< 0.001**
	Level 1 *r*_ij_		0.096	0.310			

*a = with robust standard errors*

*b = student level 1 and school level 2 sample sizes*

*c* = ***Bolded p-value ****indicates significance at the 0.05 alpha level*

#### Four CAFES measurement scales

An increase in the four-point plate scale score was significantly associated with an increase in FV served (Table 6; *p* < 0.05). This result suggests that larger, sturdier trays in a variety of colors, as well as availability of appropriate utensils, are associated with increased FV servings. All four CAFES measurement scale scores were significant predictors of FV percentage consumed (Table 7; *p* < 0.05). One percentage point increases in room, table/display, and food scale scores were associated with 0.72%, 1.34%, and 0.44% increases in FV percentage consumed, respectively (Table 7; *p* < 0.05). An increase in plate scale score was associated with a 0.24% decrease in FV percentage consumed.

**Table 6:** Predictive validity: fully conditional FV served model with four CAFES scale scores

FV SERVED^a^			Final estimation of fixed effects				
Level	Fixed Effect	*n* ^b^	*Coefficient*	*SE*	*t-ratio*	*d.f.*	*p-value* ^c^
*For Intercept, β*_*0*_	γ_00_ Intercept	*16*	44.44	144.65	0.31	9	0.77
	γ_01_% FRPM		263.21	64.86	4.06	9	**0.003**
	γ_02_% Minority		53.98	68.57	0.79	9	0.451
	γ_03_ Room scale		−97.03	147.71	−0.66	9	0.528
	γ_04_ Table/display scale		296.75	179.16	1.66	9	0.132
	γ_05_ Plate scale		122.94	47.55	2.59	9	**0.029**
	γ_06_ Food scale		28.12	64.71	0.44	9	0.674
*For Grade, β*_*1*_	γ_10_ Intercept	*1069*	7.82	16.43	0.48	1052	0.634
			Final estimation of variance components				
	Random Effect		*Variance component*	*SD*	*Χ* ^*2*^	*d.f.*	*p-value* ^c^
	Level 2 *μ*_0j_		2880.83	53.67	237.81	9	**< 0.001**
	Level 1 *r*_ij_		8287.70	91.04			

*a = with robust standard errors*

*b = student level 1 and school level 2 sample sizes*

***c*** = ***Bolded p-value ****indicates significance at the 0.05 alpha level*

**Table 7:** Predictive validity: fully conditional FV % consumed model with four CAFES scale scores

FV % CONSUMED^a^			Final estimation of fixed effects				
Level	Fixed Effect	*n* ^b^	*Coefficient*	*SE*	*t-ratio*	*d.f.*	*p-value* ^c^
*For Intercept, β*_*0*_	γ_00_ Intercept	*16*	−0.61	0.30	−2.03	9	0.073
	γ_01_% FRPM		−0.48	0.21	−2.25	9	0.051^d^
	γ_02_% Minority		−0.34	0.12	−2.83	9	**0.020**
	γ_03_ Room scale		0.72	0.21	3.48	9	**0.007**
	γ_04_ Table/display scale		1.34	0.37	3.58	9	**0.006**
	γ_05_ Plate scale		−0.24	0.05	−5.31	9	**< 0.001**
	γ_06_ Food scale		0.44	0.12	3.57	9	**0.006**
*For Grade, β*_*1*_	γ_10_ Intercept	*1011*	0.03	0.03	0.81	1052	0.416
			Final estimation of variance components				
	Random Effect		*Variance component*	*SD*	*Χ* ^*2*^	*d.f.*	*p-value* ^c^
	Level 2 *μ*_0j_		0.011	0.105	75.94	9	**< 0.001**
	Level 1 *r*_ij_		0.091	0.301			

*a = with robust standard errors*

*b = student level 1 and school level 2 sample sizes*

***c*** = ***Bolded p-value ****indicates significance at the 0.05 alpha level*

*d = Significant at the 0.10 alpha level*

The four CAFES scale scores in fully conditional models accounted for a total of 26% of the school-level variance in FV percentage consumed (Additional file 1: Table S10). A one percentage point increase in table/display scale score was associated with the largest increase in FV percentage consumed (1.34%), followed by room scale (0.72%), and food scale (0.44%). The strong association between the table/display scale was consistent with prior research findings that availability and accessibility are among the strongest predictors of dietary intake [17, 20, 23].

The negative association between plate scale score and FV percentage consumed (γ = −0.24, *p* = .03) was likely attributed to school level differences in FV offerings. Schools with higher plate scale scores -- associated with increased FV servings (Table 6) -- tended to offer more FV and allowed students to choose and serve FV themselves. The association between plate scale score and FV consumed, although not significant (γ = 26.81, *SE* = 37.22, *p* > .05), was positive indicating that students in those schools did consume more FV overall. However, students in those schools did not consume a larger percentage of the FV served when compared to schools with smaller, less sturdy trays and decreased FV offerings and choices given the significant negative association between plate scale score and FV percentage consumed (Table 7). Additional research is needed to establish whether the higher amounts of FV served or the plate scale variables contributed to this negative association.

Covariate results revealed that higher percentages of FRPM students at the school level were significantly associated with increases in FV served (Table 6; *p* < .05), but not consumed. A one percentage point increase in minority student population, however, was associated with a 0.34% reduction in FV percentage consumed (Table 7; *p* < .05). This result suggested that, although schools with higher participation in FRPM may serve more FV due to stronger wellness policies [13, 56], environmental variations captured by CAFES items, food quality, food preferences, role modeling, or nutrition education [57, 58] may contribute to lower FV percentages consumed in schools with larger percentages of minority students.

## Discussion

CAFES is the first comprehensive objective, reliable, and validated assessment tool that quantifies physical attributes of elementary school cafeterias linked to selection and consumption of FV. Internal consistency and inter-rater reliability were established across all four CAFES measurement scales, and predictive validity of FV servings and consumption was evaluated. CAFES development and testing addressed five gaps in the literature. First, although several studies have examined school food environments [2], studies addressing associations between “room scale” cafeteria design elements and eating behaviors are limited. By addressing physical attributes at multiple environmental scales, from individual food item to the design of preparation, serving, and dining areas, CAFES builds upon existing assessments that focus on, for example, nutritional aspects of the food environment [59]; economics, policy, and sociocultural factors [37]; and serving, presentation, and display items (e.g., Smarter Lunch Room Scorecards, http://smarterlunchrooms.org/resources).

Second, the predictive validity of CAFES was assessed using both FV servings and consumption data. Healthy selections are only successful if actually consumed. Environmental factors that affect food selection also differ from those that affect consumption. FV selection is affected by factors such as availability, presentation, and serving method (whether a choice is offered or not). Consumption is a function of not only choice, but also room, table/display, plate, and food scale factors [17]. Third, CAFES was validated by objective, quantitative FV servings and consumption data gathered via lunch tray photography, rather than self-report or other more subjective measures of children’s dietary intake that are unreliable [60–62]. CAFES predictive validity estimates, although small and potentially biased from missing data, are likely conservative. Because students in the predictive validity subsamples served and consumed more FV than the overall sample, schools with lower FV servings and consumption that would likely benefit most from CAFES assessment and recommended interventions were excluded from the predictive validity analysis.

Fourth, CAFES focuses on elementary school-aged children. Many food decisions, particularly for young children, occur within cafeterias. Both dietary intake and physical activity patterns established early in life likely influence long-term health [7]. Research suggests that school-based environmental interventions, such as increasing students’ FV consumption [21–24], can affect health behaviors that both reduce FV waste and set students on positive, healthy life-course trajectories [63, 64].

Fifth, CAFES focuses on elementary school cafeterias within low-income communities that often cannot implement common intervention suggestions for older children and adults targeting portion size, payment and pricing, or increasing number of meal item choices. Federally-funded meal programs regulate the portion sizes of meal items. FRPM participants who cannot afford to purchase additional items are limited to serving and consuming only the provided FRPM options. Elementary schools also typically have students pay for meals with prepaid accounts monitored by meal cards that debit meal costs in daily cafeteria lines [65]. Payment and pricing strategies, such as requiring the use of cash to pay for unhealthy items [19], cannot be used when schools do not accept cash. Furthermore, in schools with 100% of students receiving free meals, cards are used only to record students’ receipt of meals and no money is exchanged. Individual food and beverage item prices are not displayed or relevant to students’ meal selections. Moreover, not all schools offer students meal choices – a factor that affects food decisions [66] - especially when all students receive a free meal [66]. These factors render intervention suggestions related to portion size, payment and pricing, and encouraging healthy choices inapplicable to many elementary schools in low-income communities. CAFES scores, however, suggest alternative intervention strategies – many of which are low- or no-cost and can immediately be implemented - aimed at improving healthy eating among elementary school students.

## Limitations

CAFES’ limitations related to research design, FV data, and exclusion of moderating factors. CAFES development was based on a sample of elementary schools from four U.S. states with high percentages of FRPM recipients, thus findings may not generalize to other schools or regions. The cross-sectional CAFES sample also precludes causal conclusions. Limited variability among some CAFES items also affected reliability and validity estimates. CAFES also focused on lunch periods. Schools that offer USDA-funded breakfast, fruit and vegetable snack, after-school, and weekend backpack snack programs have opportunities beyond the lunch period to increase FV selection and consumption throughout the school day.

CAFES could benefit from further predictive validity analysis. The use of an objective, validated measure of FV servings and consumption is a strength of CAFES; however, the DFIA method itself – like all measures of diet – is imperfect. Measuring diet, particularly among numerous children, is notoriously difficult to do reliably and validly [44]. Even the best measures have limitations. Additional predictive validity testing is also needed to assess room and table/display subscales.

Predictive validity analyses also did not address potential school-level moderators of FV selection and consumption behavior. First, the amount of *time* students have for meals can affect selection and consumption. If students are given whole fruit that must be cut or peeled, for example, they may be less likely to select and consume that item due to the added inconvenience, difficulty, and time required [58]. Furthermore, long lines and crowded spaces, along with time pressures, can lead students to making unhealthy and impulsive selections [13]. Second, predictive validity analysis excluded social environment influences. School personnel with proper education and training can serve as role models by establishing and enforcing policies and curricula that support healthy choices [67]. The nutrition, dieting, and weight control knowledge, values, attitudes, and behaviors of teachers and other school personnel could partially account for the success or failure of healthy eating programs implemented in schools [68]. Policies and food costs that influence what schools can prepare and offer to students were also excluded from analyses. Exclusion of these moderating factors likely affected predictive validity testing; however, CAFES is intended to supplement, rather than replace, other social, cultural, economic, policy, and nutritional assessments.

## Future work

The CAFES’ tool is currently available as a paper-based assessment tool. A mobile application for Android and iOS devices is forthcoming (beta version; see CAFES.crc.nd.edu for updates or contact the corresponding author). Each require 45–90 min to complete. Paper version scoring requires an additional hour, but the mobile application automates data collection, scoring, and generating the list of intervention suggestions. These interventions, based on CAFES scoring and existing literature (e.g., how to arrange and present food to encourage healthy choices), are currently being tested and include low- and no-cost changes school staff can immediately implement.

Future CAFES work can test reducing the number of CAFES items, as well as adding other items such as kitchen, preparation, and serving area square footages and equipment inventory; objective temperature, lighting, and noise items gathered using a thermostat, lux meter, and decibel meter, respectively; and the presence of sound dampening materials to control noise. Work is also needed to establish what minimum CAFES scores are needed to achieve desired FV outcomes, such as a certain percentage increase in overall FV consumption, or to reduce the number of students not meeting USDA recommendations for daily FV intake.

Additional analyses of individual student-level moderators of the physical environment-student eating behavior relation are also needed. Student hunger level, which relates to the time of day lunch is served and whether lunch occurs before or after recess or physical education classes [69], may moderate FV selection and consumption. Additionally, student’s food perceptions and preferences should be explored. Children often make food choices based on appeal, taste, and convenience [70]. Although CAFES focused on the physical environment and improving school-level eating behaviors, these individual perceptual factors may moderate the relation between the physical environment and FV servings and consumption.

## Implications

CAFES can be used by researchers, design and public health practitioners, and school personnel to identify critical areas where environmental supports are both successful and needed, to prioritize the focus and scope of interventions, and develop low- or no-cost intervention strategies to overcome barriers to and promote healthy eating within school cafeterias. Furthermore, intervention effectiveness can be assessed by using CAFES before and after interventions are implemented. Schools can also use CAFES when developing and implementing a student wellness policy that promotes healthy eating and adequate amounts of physical activity. Since the arrangement of school cafeterias and meal items can affect students’ choices, the unintended consequences of the design and layout are important to consider. Given that school officials and food service staff do influence the types of foods that are served and how they are presented, using CAFES to establish interventions as part of the wellness policy may assist in promotion health eating among students.

## Conclusions

School cafeteria design can attract students and encourage healthy eating by becoming efficient and attractive spaces, promoting healthy eating and physical activity, and encouraging students to make healthier choices through interventions at various environmental scales [13, 15, 18, 19, 57]. Some schools have hired culinary experts to develop appealing, healthy meals and to transform cafeterias into welcoming, attractive spaces with natural lighting, artwork, and reduced noise to increase student participation in school meal programs [3, 57]. CAFES results, however, allow school staff to leverage low- or no-cost strategies, which is especially critical when facing financial constraints. CAFES proved to be a practical, easy-to-use, and inexpensive assessment tool for measuring environmental supports of and barriers to the selection and consumption of FV in elementary school cafeterias. CAFES scores, when accompanied with future intervention suggestions, will be useful in guiding school staff, researchers, nutritionists, designers, and public health policy makers in creating cafeteria environments that facilitate healthy eating. CAFES can also contribute to the development of guidelines for cafeteria design, food layout, food presentation, and other intervention strategies aimed at increasing healthy food consumption among elementary school students.

## Additional files

Additional file 1: Additional CAFES data tables. This file contains additional data tables related to CAFES development, reliability testing, and predictive validity analyses. **Table S1.** Pearson Inter-Item Correlations Among CAFES Total and Four Scale Scores. **Table S2.** Pearson Inter-Item Correlations Among CAFES Room Scale and Subscale Scores. **Table S3.** Pearson Inter-Item Correlations Among CAFES Table/Display Scale and Subscale Scores. **Table S4.** CAFES Predictive Validity Subsamples: School and Student Level Socio-Demographics. **Table S5a.** CAFES Students’ Fruit and Vegetable (FV) Servings and Percentage Consumed. **Table S5b.** Predictive Validity Subsample-CAFES Total: Student FV servings and Percentage Consumed. **Table S5c.** Predictive Validity Subsample-Four CAFES Scales: Student FV Servings and Percentage Consumed. **Table S6a.** Predictive Validity-CAFES Total Score: Fully Unconditional Model. **Table S6b.** Predictive validity-CAFES Total Score: Partially Conditional Model. **Tables S7a-b.** Predictive Validity-Four CAFES Scale Scores: Fully Unconditional Models. **Tables S8a-b.** Predictive Validity-Four CAFES Scale Scores: Partially Conditional Models. **Table S9.** Variance Accounted for by CAFES Total Score Models. **Table S10.** Variance Accounted for by Models with Four CAFES Scale Scores. (DOCX 101 kb)

Additional file 2: CAFES paper form. This file contains the paper version of the CAFES tool. (PDF 1586 kb)

Additional file 3: CAFES scoring spreadsheet. This spreadsheet file contains three worksheets. The first is the manual scoring entry spreadsheet for CAFES items. The second worksheet displays the resulting CAFES scores. The third worksheet provides a description of the CAFES scales and subscales. (XLSX 1508 kb)

## Abbreviations

BMI: Body mass index
CAFES: Cafeteria Assessment for Elementary Schools
DFIA: Digital Food Image Analysis
FRPM: Free-and reduced-price meal
FV: Fruit and vegetable
HGHY: Healthy Gardens, Healthy Youth
KR-21: Kuder-Richardson-21
USDA: United States Department of Agriculture
