# Supplementary material for: Cafeteria assessment for elementary schools (CAFES): development, reliability testing, and predictive validity analysis
Source: BMC Public Health. 2018 Oct 3;18:1154. doi: 10.1186/s12889-018-6032-2 (PMC6171137; doi:10.1186/s12889-018-6032-2)
Supplement: Supplementary file 1 — Additional CAFES data tables. This file contains additional data tables related to CAFES development, reliability testing, and predictive validity analyses. Table S1. Pearson Inter-Item Correlations Among CAFES Total and Four Scale Scores. Table S2. Pearson Inter-Item Correlations Among CAFES Room Scale and Subscale Scores. Table S3. Pearson Inter-Item Correlations Among CAFES Table/Display Scale and Subscale Scores. Table S4. CAFES Predictive Validity Subsamples: School and Student Level Socio-Demographics. Table S5a. CAFES Students’ Fruit and Vegetable (FV) Servings and Percentage Consumed. Table S5b. Predictive Validity Subsample-CAFES Total: Student FV servings and Percentage Consumed. Table S5c. Predictive Validity Subsample-Four CAFES Scales: Student FV Servings and Percentage Consumed. Table S6a. Predictive Validity-CAFES Total Score: Fully Unconditional Model. Table S6b. Predictive validity-CAFES Total Score: Partially Conditional Model. Tables S7a-b. Predictive Validity-Four CAFES Scale Scores: Fully Unconditional Models. Tables S8a-b. Predictive Validity-Four CAFES Scale Scores: Partially Conditional Models. Table S9. Variance Accounted for by CAFES Total Score Models. Table S10. Variance Accounted for by Models with Four CAFES Scale Scores. (DOCX 101 kb) [file 12889_2018_6032_MOESM1_ESM.docx]

**Additional File 0-Additional CAFES Tables**

**Table S1. Pearson Inter-Item Correlations among CAFES Total and Four Scale Scores**

| ***Score*** | | ***1*** | ***2*** | ***3*** | ***4*** | ***5*** |
| --- | --- | --- | --- | --- | --- | --- |
| **1.** | **CAFES score**  *(n)* | -- | .64^**^ | .83^**^ | .37* | -.33 |
|  |  |  | *32* | *36* | *30* | *20* |
| **2.** | **Room scale**  *(n)* |  | -- | .14 | .13 | .02 |
|  |  |  |  | *32* | *29* | *19* |
| **3.** | **Table/Display scale**  *(n)* |  |  | -- | .15 | -.47* |
|  |  |  |  |  | *30* | *20* |
| **4.** | **Plate scale**  *(n)* |  |  |  | -- | -.17 |
|  |  |  |  |  |  | *26* |
| **5.** | **Food scale**  *(n)* |  |  |  |  | -- |
|  |  |  |  |  |  |  |

** = p< 0.05 level (two-tailed) ** = p< 0.01 level (two-tailed)*

Average correlation = .34

**Table S2. Pearson Inter-Item Correlations among CAFES Room Scale and Subscale Scores**

| ***SCALE/subscale*** | | ***1*** | ***2*** | ***3*** | ***4*** | ***5*** | ***6*** | ***7*** |
| --- | --- | --- | --- | --- | --- | --- | --- | --- |
| **1.** | **ROOM SCALE**  *(n)* | -- | .19 | .48** | .43* | .41* | -.21 | .59** |
|  |  |  | *25* | *32* | *31* | *32* | *32* | *31* |
| **2.** | **Ambient**  *(n)* |  | -- | -.01 | -.05 | .31 | -.50* | -.25 |
|  |  |  |  | *24* | *25* | *24* | *25* | *23* |
| **3.** | **Appearance**  *(n)* |  |  | -- | .23 | -.13 | -.10 | .19 |
|  |  |  |  |  | *30* | *31* | *31* | *30* |
| **4.** | **Windows**  *(n)* |  |  |  | -- | -.26 | .04 | -.16 |
|  |  |  |  |  |  | *30* | *31* | *29* |
| **5.** | **Layout**  *(n)* |  |  |  |  | -- | -.13 | .12 |
|  |  |  |  |  |  |  | *31* | *31* |
| **6.** | *(Healthy Signage***)*  *(n)* |  |  |  |  |  | -- | -.19 |
|  |  |  |  |  |  |  |  | *30* |
| **7.** | **Kitchen/Serving**  *(n)* |  |  |  |  |  |  | -- |
|  |  |  |  |  |  |  |  |  |

** = p< 0.05 level (two-tailed) ** = p< 0.01 level (two-tailed)*

**** = This “subscale” only included one item*

Average correlation = .22

**Table S3. Pearson Inter-Item Correlations Among**

**CAFES Table/Display Scale and Subscale Scores**

| ***SCALE/subscale*** | | ***1*** | ***2*** | ***3*** | ***4*** | ***5*** | ***6*** |
| --- | --- | --- | --- | --- | --- | --- | --- |
| **1.** | **TABLE/DISPLAY SCALE**  *(n)* | -- | 0.56** | 0.79** | 0.08 | 0.12 | 0.53** |
|  |  |  | *27* | *33* | *27* | *25* | *33* |
| **2.** | **Furniture**  *(n)* |  | -- | 0.38 | -0.09 | -0.15 | 0.29 |
|  |  |  |  | *27* | *28* | *26* | *27* |
| **3.** | **Availability**  *(n)* |  |  | -- | -0.26 | 0.02 | 0.03 |
|  |  |  |  |  | *27* | *25* | *33* |
| **4.** | **Display**  *(n)* |  |  |  | -- | 0.41* | -0.03 |
|  |  |  |  |  |  | *26* | *27* |
| **5.** | **Serving Method**  *(n)* |  |  |  |  | -- | -0.33 |
|  |  |  |  |  |  |  | *25* |
| **6.** | **Variety**  *(n)* |  |  |  |  |  | -- |
|  |  |  |  |  |  |  |  |

** = p< 0.05 level (two-tailed) ** = p< 0.01 level (two-tailed)*

Average correlation = .27

**Table S4. CAFES Predictive Validity Subsamples: School and Student Level Socio-Demographics**

|  | | **CAFES TOTAL + outcome data**^a^ | | | |  | **4 CAFES scales + outcome data**^b^ | | | |
| --- | --- | --- | --- | --- | --- | --- | --- | --- | --- | --- |
| **Variable** | ***Data Level*** | **n**  *(tot)* | **Variable**  **Levels** | **Total** | |  | **n**  *(tot)* | **Levels** | **Total** | |
|  |  |  |  | # | % |  |  |  | # | % |
| **Location** | *School* | 29 | Arkansas  Iowa  New York  Washington | 6  6  13  4 | 21  21  45  13 |  | 16 | Arkansas  Iowa  New York  Washington | 2  4  7  3 | 12  25  44  19 |
| **Urbanity** | *School* | 29 | Urban  Rural  Suburban | 9  15  5 | 31  52  17 |  | 16 | Urban  Rural  Suburban | 6  5  5 | 38  31  31 |
| **Gender** | *Student* | 856  *(1544)* | Male  Female  *Missing* | *403*  *453*  *688* | 26  29  *45* |  | 595  *(1069)* | Male  Female  *Missing* | 294  301  *474* | 28  28  44 |
| **Free & reduced price meal recipients** | *Student* | 848  *(1544)* | Full  Reduced  Free  *Missing* | 201  95  552  *696* | 13  6  36  *45* |  | 606  *(1069)* | Full  Reduced  Free  *Missing* | 139  63  404  *463* | 13  6  38  *43* |
| **Grade** | *Student* | 1544 | 2^nd^  4^th^/5^th^ | 724  820 | 47  53 |  | 1069 | 2^nd^  4^th^/5^th^ | 520  549 | 49  51 |
| **Ethnicity** | *Student* | 857  *(1544)* | White  Black  Hispanic  Asian  Native American  Other  *Missing* | 365  201  172  26  13  80  *687* | 24  13  11  2  1  5  44 |  | 612  *(1069)* | White  Black  Hispanic  Asian  Native American  Other  *Missing* | 223  136  153  20  12  68  *457* | 21  13  14  2  1  6  43 |
|  |  |  |  |  | |  |  |  |  |  |
| **Student population**  *(# students)* | *School* | 29 | Mean  SD  Range | 419  182  120 – 894 | |  | 16 | Mean  SD  Range | 440  169  166 – 894 | |
| **% Free & reduced price meal recipients** | *School* | 29 | Mean  SD  Range | 68%  19%  43 – 100% | |  | 16 | Mean  SD  Range | 65%  16%  43 – 99% | |
| **Ethnicity**  *(% minority students)* | *School* | 29 | Mean  SD  Range | 54%  33%  1 – 99% | |  | 16 | Mean  SD  Range | 61%  27%  8 – 98% | |
| **BMI** | *Student* | 373  *(1544)* | Mean  SD  Range  *Missing* | 19.7  5.4  11.4 – 46.4  *1171* | |  | 254  *(1069)* | Mean  SD  Range  *Missing* | 19.6  5.4  11.4 – 46.4  *815* | |
| **Age** | *Student* | 851  *(1544)* | Mean  SD  Range  *Missing* | 8.4  1.2  6 – 12  *693* | |  | 590  *(1069)* | Mean  SD  Range  *Missing* | 8.4  1.2  6 – 12  *479* | |

*a= Schools that provided FV lunch tray outcome data and at least 50% of all CAFES items*

*b= Schools that provided FV lunch tray outcome data and at least 50% of all four CAFES scale items*

**Table S5a. CAFES Students’ Fruit and Vegetable (FV) Servings and Percentage Consumed**

|  | **N**  Schools | **N**  Students | **FV served** *(grams)* | | | **n*** | **FV percentage consumed** | | |
| --- | --- | --- | --- | --- | --- | --- | --- | --- | --- |
|  |  |  | **Mean (*SD*)** | | **Range** |  | **Mean (SD)** | | **Range** |
| Fruits | 44 | 2506 | 102.38 | *(87.40)* | 0 – 522.24 | 2024 | 65% | (*37%*) | 0 - 100% |
| Vegetables | 44 | 2506 | 42.02 | *(49.17)* | 0 - 514.10 | 1799 | 57% | (*38%*) | 0 - 100% |
| **Total FV** | 44 | 2506 | 145.61 | *(109.95)* | 0 - 1028.19 | 2314 | 63% | (*34%*) | 0 - 100% |

** = “FV percentage consumed” is reported only for students who served or were served more than 0 grams of fruits and vegetables*

**S5b Table. Predictive Validity Subsample 1-CAFES Total: Student FV servings and Percentage Consumed.**

|  | **n**  Schools | **n**  Students | **FV served** *(grams)* | | | **n*** | **FV percentage consumed** | | |
| --- | --- | --- | --- | --- | --- | --- | --- | --- | --- |
|  |  |  | **Mean (*SD*)** | | **Range** |  | **Mean (SD)** | | **Range** |
| Fruits | 29 | 1544 | 117.75 | *(91.91)* | 0 – 522.24 | 1316 | 65% | *(37%)* | 0 - 100% |
| Vegetables | 29 | 1544 | 45.02 | *(52.41)* | 0 - 514.10 | 1112 | 55% | *(38%)* | 0 - 100% |
| **Total FV** | 29 | 1544 | 164.45 | *(119.61)* | 0 - 1028.19 | 1441 | 63% | *(34%)* | 0 - 100% |

** = “FV percentage consumed” is reported only for students who served or were served more than 0 grams of fruits and vegetables*

**S5c Table. Predictive Validity Subsample 2-Four CAFES Scales: Student FV Servings and Percentage Consumed.**

|  | **n**  Schools | **n**  Students | **FV served** *(grams)* | | | **n*** | **FV percentage consumed** | | |
| --- | --- | --- | --- | --- | --- | --- | --- | --- | --- |
|  |  |  | **Mean (*SD*)** | | **Range** |  | **Mean (SD)** | | **Range** |
| Fruits | 16 | 1069 | 117.61 | *(64.32)* | 0 – 522.24 | 927 | 63% | *(37%)* | 0 - 100% |
| Vegetables | 16 | 1069 | 45.64 | *(45.77)* | 0 - 514.10 | 857 | 52% | *(37%)* | 0 - 100% |
| **Total FV** | 16 | 1069 | 165.26 | *(111.77)* | 0 - 1028.19 | 1011 | 60% | *(34%)* | 0 - 100% |

** = “FV percentage consumed” is reported only for students who served or were served more than 0 grams of fruits and vegetables*

*Note that sample sizes for the “FV percentage consumed” outcomes in S5a-c Tables were smaller because only students who were served or served themselves more than zero grams of FV could be included in those analyses. Students who did not consume any fruits or vegetables were included in analyses of FV served.*

S5a-c Tables contain predictive validity subsample sample sizes and a descriptive summary of fruit, vegetable, and FV served and percent consumed outcomes. S5a Table displays FV outcome summary statistics for the entire sample of 44 schools that collected lunch tray photography data. S5b Table summarizes FV outcome variables for the 29-school sample that provided at least 50% of all CAFES data, and S5c Table contains FV outcome summaries for the 16 schools that provided at least 50% of all items in each of the four CAFES scales.

**Table S6a. Predictive Validity-CAFES Total Score: Fully Unconditional Model**

| **FV % CONSUMED** |  | **Final estimation of fixed effects**  *(with robust standard errors)* | | | | |
| --- | --- | --- | --- | --- | --- | --- |
| **Fixed Effect** | *n*^a^ | *Coefficient* | *SE* | *t-ratio* | *d.f.* | *p-value*^b^ |
| Mean %FV consumed, γ_00_ | 29 | 0.644 | 0.026 | 24.52 | 28 | **<0.001** |
|  |  | **Final estimation of variance components** | | | | |
| **Random Effect** |  | *Variance component* | *SD* | *Χ^2^* | *d.f.* | *p-value*^b^ |
| Level 2 *μ*_0j_ |  | 0.018 | 0.135 | 318.26 | 28 | **<0.001** |
| Level 1 *r*_ij_ | 1544 | 0.096 | 0.310 |  |  |  |

***a****= student level 1 and school level 2 sample sizes*

***b*= *Bolded p-value*** *indicates significance at the 0.05 alpha level*

**Table S6b. Predictive validity-CAFES Total Score: Partially Conditional Model**

| **FV % CONSUMED** |  | **Final estimation of fixed effects**  *(with robust standard errors)* | | | | |
| --- | --- | --- | --- | --- | --- | --- |
| **Fixed Effect** | *n*^a^ | *Coefficient* | *SE* | *t-ratio* | *d.f.* | *p-value*^b^ |
| Mean %FV consumed, γ_00_ | 29 | 0.644 | 0.025 | 25.91 | 26 | **<0.001** |
| γ_01_ % FRPM |  | -0.190 | 0.194 | -0.98 | 26 | 0.335 |
| γ_02_ % Minority |  | -0.051 | 0.094 | -0.55 | 26 | 0.590 |
|  |  | **Final estimation of variance components** | | | | |
| **Random Effect** |  | *Variance component* | *SD* | *Χ^2^* | *d.f.* | *p-value*^b^ |
| Level 2 *μ*_0j_ |  | 0.017 | 0.132 | 280.18 | 26 | **<0.001** |
| Level 1 *r*_ij_ | 1441 | 0.096 | 0.310 |  |  |  |

***a****= student level 1 and school level 2 sample sizes*

***b*= *Bolded p-value*** *indicates significance at the 0.05 alpha level*

**Table S7a-b. Predictive Validity-Four CAFES Scale Scores: Fully Unconditional Models**

| **a. FV SERVED** | | **Final estimation of fixed effects**  *(with robust standard errors)* | | | | |
| --- | --- | --- | --- | --- | --- | --- |
| **Fixed Effect** | *n*^a^ | *Coefficient* | *SE* | *t-ratio* | *d.f.* | *p-value^b^* |
| Mean FV served, γ_00_ | 16 | 166.32 | 15.92 | 10.45 | 15 | **<0.001** |
|  |  | **Final estimation of variance components** | | | | |
| **Random Effect** |  | *Variance component* | *SD* | *Χ^2^* | *d.f.* | *p-value^b^* |
| Level 2 *μ*_0j_ |  | 4173.35 | 64.60 | 556.39 | 15 | **<0.001** |
| Level 1 *r*_ij_ | 1069 | 8290.81 | 91.05 |  |  |  |

| **b. FV % CONSUMED** |  | **Final estimation of fixed effects**  *(with robust standard errors)* | | | | |
| --- | --- | --- | --- | --- | --- | --- |
| **Fixed Effect** | *n*^a^ | *Coefficient* | *SE* | *t-ratio* | *d.f.* | *p-value^b^* |
| Mean %FV consumed, γ_00_ | 16 | 0.598 | 0.037 | 16.11 | 15 | **<0.001** |
|  |  | **Final estimation of variance components** | | | | |
| **Random Effect** |  | *Variance component* | *SD* | *Χ^2^* | *d.f.* | *p-value^b^* |
| Level 2 *μ*_0j_ |  | 0.022 | 0.148 | 258.84 | 15 | **<0.001** |
| Level 1 *r*_ij_ | 1011 | 0.091 | 0.301 |  |  |  |

***a****= student level 1 and school level 2 sample sizes*

***b*= *Bolded p-value*** *indicates significance at the 0.05 alpha level*

**Table S8a-b. Predictive Validity-Four CAFES Scale Scores: Partially Conditional Models**

| **a. FV SERVED** | | | **Final estimation of fixed effects**  *(with robust standard errors)* | | | | |
| --- | --- | --- | --- | --- | --- | --- | --- |
| **Level** | **Fixed Effect** | *n*^a^ | *Coefficient* | *SE* | *t-ratio* | *d.f.* | *p-value*^b^ |
| *For Intercept, β_0_* | γ_00_ Intercept | *16* | 97.84 | 10.81 | 9.05 | 13 | **<0.001** |
|  | γ_01_ % FRPM |  | *12.55* | 65.37 | 0.19 | 13 | 0.851 |
|  | γ_02_ % Minority |  | -27.60 | 32.77 | -0.84 | 13 | 0.415 |
| *For Grade, β_1_* | γ_10_ Intercept | *1069* | 11.17 | 11.68 | 0.96 | 1052 | 0.339 |
|  |  | | **Final estimation of variance components** | | | | |
|  | **Random Effect** |  | *Variance component* | *SD* | *Χ^2^* | *d.f.* | *p-value*^b^ |
|  | Level 2 *μ*_0j_ |  | 2150.25 | 46.37 | 277.29 | 13 | **<0.001** |
|  | Level 1 *r*_ij_ |  | 7128.36 | 84.43 |  |  |  |

| **b. FV % CONSUMED** | | | **Final estimation of fixed effects**  *(with robust standard errors)* | | | | |
| --- | --- | --- | --- | --- | --- | --- | --- |
| **Level** | **Fixed Effect** | *n*^a^ | *Coefficient* | *SE* | *t-ratio* | *d.f.* | *p-value*^b^ |
| *For Intercept, β_0_* | γ_00_ Intercept | *16* | 0.598 | 0.031 | 19.33 | 13 | **<0.001** |
|  | **γ_01_ % FRPM** |  | **-0.522** | **0.221** | **-2.37** | **13** | **0.034** |
|  | γ_02_ % Minority |  | -0.030 | 0.111 | -0.27 | 13 | 0.791 |
| *For Grade, β_1_* | γ_10_ Intercept | *1011* | 0.030 | 0.031 | 0.95 | 1052 | 0.345 |
|  |  | | **Final estimation of variance components** | | | | |
|  | **Random Effect** |  | *Variance component* | *SD* | *Χ^2^* | *d.f.* | *p-value*^b^ |
|  | Level 2 *μ*_0j_ |  | 0.017 | 0.130 | 170.16 | 13 | **<0.001** |
|  | Level 1 *r*_ij_ |  | 0.091 | 0.301 |  |  |  |

***a****= student level 1 and school level 2 sample sizes*

***b*= *Bolded p-value*** *indicates significance at the 0.05 alpha level*

**Table S9 Variance Accounted for by CAFES Total Score Models**

|  | **Incremental variance: Partially Conditional**^a^ | | **Incremental variance:**  **Fully conditional**^b^ | | **Total variance**  **accounted for**^c^ | |
| --- | --- | --- | --- | --- | --- | --- |
| **Outcome** | **% Within**  s*tudent level* | **% Between**  *school level* | **% Within**  s*tudent level* | **% Between**  *school level* | **% Within**  s*tudent level* | **% Between**  *school level* |
| **% FV consumed** | 0.06 | 0.02 | 0.00 | 13.11 | 0.06 | 17.77 |

*a= Percent variance accounted for by controls: student grade level; % FRPM and % minority populations*

*b= Percent variance accounted for by room, table, plate, and food scale scores*

*c= Percent of the total variance accounted for by the fully conditional model (controls & predictors)*

**Table S10. Variance Accounted for by Models with Four CAFES Scale Scores.**

|  | **Incremental variance: Partially Conditional**^a^ | | **Incremental variance:**  **Fully conditional**^b^ | | **Total variance**  **accounted for**^c^ | |
| --- | --- | --- | --- | --- | --- | --- |
| **Outcome** | **% Within**  s*tudent level* | **% Between**  *school level* | **% Within**  s*tudent level* | **% Between**  *school level* | **% Within**  s*tudent level* | **% Between**  *school level* |
| FV served | 0.05 | 9.35 | -0.01 | 21.62 | 0.04 | 30.97 |
| **% FV consumed** | 0.11 | 22.78 | -0.03 | 26.26 | 0.08 | 49.04 |

*a = Percent variance accounted for by controls: student grade level; % FRPM and % minority populations*

*b = Percent variance accounted for by room, table, plate, and food scale scores*

*c = Percent of the total variance accounted for by the fully conditional model (controls & predictors)*

*A negative percent variance indicates an inverse relationship between predictors and outcomes.*
