# Supplementary material for: Cafeteria assessment for elementary schools (CAFES): development, reliability testing, and predictive validity analysis
Source: BMC Public Health. 2018 Oct 3;18:1154. doi: 10.1186/s12889-018-6032-2 (PMC6171137; doi:10.1186/s12889-018-6032-2)
Supplement: Supplementary file 2 — CAFES paper form. This file contains the paper version of the CAFES tool. (PDF 1586 kb) [file 12889_2018_6032_MOESM2_ESM.pdf]

**INSTRUCTIONS: The CAFES tool evaluates 2nd-5th grade elementary school cafeterias and students. Please complete the following items as noted. Use the UNCODED VERSION for observations; refer to the CODED VERSION to score CAFES.**

**= when possible, complete item *just before/during* a meal time** **= DO NOT complete item during a meal time**

**SUPPLY LIST:** *CAFES forms, Camera (batteries/memory cards), Ruler & tape measure, writing utensils & extra paper*

**A0. Please enter the following information about the CAFES tool user and the observed school.**

**A1-2. CAFES user name**: ________________________________ **A3. School name**: __________________________________

**A4. School street address/city:** ___________________________________________ **A5. State:** ________ **A6. Zip:** ___________

**A7. Participating Grades** (circle all that apply): 2nd 3rd 4th 5th **A8. CAFES Observation Date:** _____ / _______ / _______

**A9. Is this observation being completed during a lunch period?** ☐ Yes ☐ No

**A10. Is this observation being completed while lunch food is present in the serving area?** ☐ Yes ☐ No

**B0. Please ask Food Service staff and/or the Principal the following questions.**

**B1. How many cafeterias/eating areas are used for student lunch?** *If more than one cafeteria or eating area is used, evaluate the space used by the participating grades, or complete separate CAFES evaluations for each space.* __________

**B2. Select the grade levels that use the eating area being evaluated by CAFES:** ☐ 2nd ☐ 3rd ☐ 4th ☐ 5th

| No. | Question: | Yes | No | Additional response |
|---|---|---|---|---|
| B3.1 | Are food or beverage vending machines available for student use during lunch? *If no, select N/A for B3.1b.* | ☐ | ☐ | **B3.1a** # Machines:______ |
| B3.1b | *If yes,* are only fruits and vegetables available? | ☐ | ☐ | ☐ N/A |
| B3.2 | Do fundraisers involving food occur DURING LUNCH TIME? | ☐ | ☐ | |
| B3.3 | Do fundraisers involving food occur IN THE CAFETERIA OR EATING AREA(S) during lunch time? | ☐ | ☐ | |
| B3.4 | Are different portion sizes available for students of different ages/grades (K–3rd / 4th-6th / 7th+)? | ☐ | ☐ | |
| B3.5 | Are school lunches prepared by a contracted/outside food company? | ☐ | ☐ | |
| B3.6 | What is the total student population? ___________________ *students* | | | **B3.10 Length of each period:** ____________________ *minutes* |
| B3.7 | How many students eat per LUNCH period? ______________ *students/period* | | | |
| B3.8 | How many lunch periods occur per day? _________________ *periods* | | | |
| B3.9 | Note the start and end times of each lunch period: ____________________________________________________ | | | |

**C0. Please ask Food Service staff the following questions.**

**C1. Are meals prepared (not just reheated or assembled) at the school?** ☐ Yes ☐ No

**C2. Considering the last year, which of the following factors HELPED your school cafeteria's ability to provide healthier food choices? Select all that apply.**

**a.** ☐ Suitable equipment available **b.** ☐ Adequate storage space available **c.** ☐ Neither

**C3A. Considering the last year, which of the following factors HELPED your school cafeteria's ability to provide healthier food choices? Select all that apply.**

**a.** ☐ Suitable preparation area available **b.** ☐ Adequate display space available **c.** ☐ Neither

**C3B. Considering the last year, did a lack of storage space LIMIT your school cafeteria's ability to provide healthier food choices?** ☐ Yes ☐ No

**Observer name**: ________________________________ **State** _________ **School ID#**_________________

**C4. Students serve themselves for (check all that apply):**

**a.** ☐ A la carte lunch entrees **b.** ☐ A la carte lunch sides **c.** ☐ Salad/veggies/fruit **d.** ☐ None of the above

**C5. Is the "offer-versus-serve" option used during lunch (students are asked whether or not they want a particular meal item) for any of the following items? Check all that apply.**

**a.** ☐ A la carte lunch entrees **b.** ☐ A la carte lunch sides **c.** ☐ Salad/veggies/fruit **d.** ☐ None of the above

| **D0.** ***Please ask Food Service staff how often each of the following items are offered during lunch, on average, each week and check the average number of days per 5-day week.*** | | **Average days per week** | | | | | |
|---|---|---|---|---|---|---|---|
| | | **0** | **1** | **2** | **3** | **4** | **5** |
| **D1** | Food reheated for students' lunches | | | | | | |
| **D2** | TWO or more different entrees/main courses | | | | | | |
| **D3** | TWO or more different vegetables | | | | | | |
| **D4** | TWO or more different fruits | | | | | | |
| **D5.1** | Salad | | | | | | |
| **D5.2** | *Note if salad is an entrée, side, or both (or n/a)*: _______________________________ | | | | | | |
| **D6** | Whole grains | | | | | | |
| **D7** | Pizza | | | | | | |
| **D8** | French fried or *breaded* potatoes, hash browns, tater tots, including baked/reheated | | | | | | |
| **D9** | Spaghetti, macaroni, or other pasta | | | | | | |
| **D10** | Cookies, crackers, chips, pastries, cakes, other baked goods ***not*** low in fat | | | | | | |
| **D11** | Low fat or fat free cookies, crackers, chips, pastries, cakes, other baked goods | | | | | | |
| **D12** | Ice cream or frozen yogurt ***not*** low in fat | | | | | | |
| **D13** | Low fat or fat free ice cream, frozen yogurt, sherbet, slushy, icy | | | | | | |
| **D14** | A la carte lunch | | | | | | |

**E0. Please ask Food Service staff what beverages are available to students during lunch.**

**E1. What type of milk is available for students to purchase during lunch? Check all that apply.**

**a.** ☐ Whole/full-fat UNFLAVORED milk and/or soy milk

**b.** ☐ Whole/full-fat FLAVORED milk and/or soy milk

**c.** ☐ Reduced/low-fat, skim, or fat-free UNFLAVORED white and/or soy milk

**d.** ☐ Reduced/low-fat, skim, or fat-free FLAVORED milk and/or soy milk

**e.** ☐ ONLY reduced/low-fat, skim, or fat-free milk available (including white, flavored, and soy milks)

**f.** ☐ ONLY UNFLAVORED reduced/low-fat, skim, or fat-free milk available (including white and soy milks)

**g.** ☐ No milk is available for students to purchase during lunch

**E2. Are juice and other sweetened beverages available to students during lunch?**

**a.** ☐ Yes, but only 100% juice **b.** ☐ Yes, <100% juice and other sweetened beverages **c.** ☐ No

**E3. Is water available to students during lunch (not just a drinking fountain without cups)?** ☐ Yes ☐ No

Observer name: ______________________________ State _________ School ID#________________

## F0. Cafeteria Ambient Environment

**Please rate the following for the CAFETERIA/EATING AREA.** Remember to complete separate CAFES evaluations if there are multiple cafeterias/eating areas.

| | Great | Good | Fair | Poor | Other |
|---|---|---|---|---|---|
| **F1. TEMPERATURE** | ☐ OK | | ☐ Hot or cold | | |
| **F2. Is AIR CONDITIONING available?** | ☐ Yes | | ☐ No A/C | | |
| **F3.2. LIGHTING** | ☐ Bright/Adequately lit | | ☐ Dark (*if lights are off, select N/A*) | | ☐ N/A lights off |
| **F4.2. ODOR** | ☐ No smell or pleasant smell | ☐ Noticeable but not unpleasant odor | ☐ Slightly unpleasant odor | ☐ Strong, unpleasant odor | ☐ Food is present during observation of odor |
| **F5.2. NOISE** | ☐ Very quiet | ☐ Soft voices | ☐ Loud talking | ☐ Yelling/screaming | ☐ N/A no students |
| **F6. MUSIC during lunch?** | ☐ Yes | | ☐ No | | |

## G0. Cafeteria Appearance

**Please rate the following for the CAFETERIA/EATING AREA** (complete separate CAFES forms for each area)**.**

| | Great | Good | Fair | Poor | Other |
|---|---|---|---|---|---|
| **G1. Attractiveness** | ☐ Good physical condition, bright, clean, child appropriate | | ☐ Poor physical condition, dark, dirty, not child appropriate | | |
| **G2. Physical structure** *(floors, walls, ceiling)* | ☐ Clean, well kempt, no damage or cracks | ☐ Stained but clean; no repair needed | ☐ Some damage, cracks, peeling paint | ☐ Unkempt, dirty, peeling paint, leaks, damaged | |
| **G3. Clutter** | ☐ No clutter | ☐ Almost no clutter | ☐ Some clutter | ☐ Max. clutter/chaos | |
| **G4. Cleanliness** | ☐ Clean | ☐ Almost clean | ☐ Satisfactory | ☐ Dirty/moldy | |
| **G5.2. Condition of tables and seating** | ☐ Clean, no damage | ☐ Stained but clean; no damage | ☐ Stains, needs some repairs and paint | ☐ Dirty, damaged, paint needed | ☐ Not present |
| **G6.2. Attractiveness of tables & seating** | ☐ Good physical condition, bright, clean, child appropriate | | ☐ Poor physical condition, dark, dirty, not child appropriate | | ☐ Not present |

## H. Cafeteria Layout

**Are any of the following items visible from the cafeteria/eating area?**

**H1. Food or beverages** ☐ Yes, all ☐ No, all in separate room ☐ Only fruit, vegetables, whole grains, skim white milk ☐ Only other items

**H2. Food vending machines** ☐ Yes ☐ No

**H3. Beverage vending machines** ☐ Yes ☐ No

**H4. Observe student circulation (path from the entrance to serving area, seating, trash, and exit) during lunch, or ask food service staff if the student circulation is:**

☐ Unclear: lots of overlapping paths and areas for congestion

☐ Clear: no/only minor overlapping: enter, food, seating, trash, exit without interfering paths or chaos

**H5. Are there any obstructions in the cafeteria/eating or serving areas that affect student movement (e.g., columns, piers, pipes)?** ☐ Yes ☐ No

**Observer name**: ________________________________ **State** _________ **School ID#**_________________

**I. Please follow the instructions to calculate the area of the cafeteria/eating area.** If your school has more than one cafeteria/eating area for elementary students, complete separate CAFES forms for each space.

**I1. Using the space below, please draw the shape of the cafeteria/eating area floor (e.g., square, rectangle, L-shaped, etc.). Measure the length of each cafeteria wall in feet. On the drawing, label each wall with the appropriate measurement. Please convert all measurements to feet, up to two decimal places (9 ft - 3 in = 9.25 ft).**

*I1A. If your school is participating in a CAFES research study, please take a picture of the drawing you just created and share it with the researchers.*

**I2. Calculate the total cafeteria floor area in square feet (SF), up to two decimal places, and enter the result.**
For example, a 20' x 10'-4" (10.33) rectangular space has an area of 206.67 SF. ____________________________________
*(area formulas: square/rectangle = Length x Width; triangle = ½ Base x Height; circle = 3.14 x radius$^2$) The area of a triangular space is calculated using the formula 1/2(base x height) where the base and height are entered in square feet (SF), up to two decimal places. The area of a circular space is 3.14 x radius x radius.*

**I3. Measure the highest and lowest ceiling heights** *(feet, inches)*. If ceiling heights are too high to measure, measure one cinder block, brick, or wall tile and count those, or estimate the height. Convert measurements to feet, up to two decimal places (e.g., enter 9.5 for a 9'-6" ceiling; enter 9.25 for a 9'-3" ceiling), and enter here:

**I3.1. Ceiling height, high: ___________________ FT** **I3.2. Ceiling height, low: ___________________ FT**

**I4. Calculate the total cafeteria wall area in feet, up to two decimal places. For each wall, multiply its length and height to calculate its area, then sum the areas of all walls. Enter the sum here: _____________________________________ FT**

**J0. Please follow the instructions to calculate the area of windows present in the cafeteria/eating area.**

**J1. Does the cafeteria/eating area have windows?** ☐ **Yes** *(continue to J2)* ☐ **No** *(skip to section K.)*

**J2. Cafeteria Window Area. Measure each window height** *(h)* **and width** *(w)* **in feet (two decimal places).** Measure the window glass from top to bottom (h) and left to right (w). Ignore any divisions in the glass within a single window.

| **Size** | ***Height*** (ft) x | **Width** (ft) x | **Quantity** = | **Area Total** | **Size** | ***Height*** (ft) x | **Width** (ft) x | **Quantity** = | **Area Total** |
|---|---|---|---|---|---|---|---|---|---|
| 1 | _________ | _________ | _________ | __________ | 7 | _________ | _________ | _________ | __________ |
| 2 | _________ | _________ | _________ | __________ | 8 | _________ | _________ | _________ | __________ |
| 3 | _________ | _________ | _________ | __________ | 9 | _________ | _________ | _________ | __________ |
| 4 | _________ | _________ | _________ | __________ | 10 | _________ | _________ | _________ | __________ |
| 5 | _________ | _________ | _________ | __________ | 11 | _________ | _________ | _________ | __________ |
| 6 | _________ | _________ | _________ | __________ | 12 | _________ | _________ | _________ | __________ |

**J3. Total window area**: For each window type, calculate the total area in SF (H x W X Quantity), then sum all window areas and enter the sum here in square feet, up to two decimal places.: ______________________________________________________

Observer name: ___________________________________ State ______________ School ID#________

K0. Cafeteria Furniture

**K1. The following questions ask about the tables and seating in the student cafeteria/eating area.**

| | |
|---|---|
| **K2.1 How many tables are RECTANGULAR with ATTACHED seating?** | # |
| **K2.2 How many tables are RECTANGULAR with DETACHED chairs?** | # |
| **K2.3 How many tables are CIRCULAR/SQUARE with ATTACHED seating?** | # |
| **K2.4 How many tables are CIRCULAR/SQUARE with DETACHED chairs?** | # |
| **K3.1 Enter the total number of attached individual seats:** | # |
| **K3.2 Enter the total number of detached individual seats:** | # |
| **K3.3 Enter the total number of attached benches:** | # |

| | | | |
|---|---|---|---|
| **K4. Do students sit on individual seats or benches?** | ☐ Benches | ☐ Individual Seats | ☐ Both |
| **K5. Are seats attached to the tables?** | ☐ Yes | ☐ No | ☐ Both |
| **K6. Is there a "sharing" table where students can leave or take uneaten food items?** | ☐ Yes | ☐ No | |
| **K7. Table shapes are** | ☐ All rectangular | ☐ Some or all are circular/square | |

Cafeteria Windows

**L0. Please complete the following items about cafeteria/eating area WINDOWS on exterior walls.**

| Item | Response | Response | Response | N/A |
|---|---|---|---|---|
| **L1. Does the cafeteria/eating area have WINDOWS/SKYLIGHTS?** | ☐ Yes (includes skylights) | ☐ Interior windows only | ☐ No *(check N/A for L2-L7)* | |
| **L2. Rate the condition of the windows** *(not skylights).* | ☐ Clean, not broken or damaged, transparent; only minor cleaning, painting, or repairs needed | ☐ Dirty, cracked, damaged, or broken; peeling paint; not transparent | ☐ No windows/only skylights | ☐ N/A |
| **L3. How natural *(trees, grass, water)* is the view from the windows?** | ☐ More than ½ natural | ☐ Less than ½ natural | ☐ No view/only non-natural interior views | ☐ N/A |
| **L4. Are the windows operable?** | ☐ All, some | ☐ None/interior windows only | | ☐ N/A |
| **L5. Are the windows transparent** *(i.e., not tinted, opaque, or too dirty to see through)?* | ☐ All, some | ☐ None are transparent | | ☐ N/A |
| **L6. Do windows have treatments such as blinds, curtains, or shades to control light?** | ☐ All, some | ☐ No treatments | | ☐ N/A |
| **L7. Do the windows have screens?** | ☐ All, some | ☐ No screens/interior windows only | | ☐ N/A |

Observer name: ______________________________ State ____________ School ID#________

**M0. Advertisements – Cafeteria and serving areas**

**Inside the CAFETERIA/EATING and SERVING AREA, are advertisements, signs, and/or flyers visible for:**

| Topic | Yes | No | Quantity # *(If no, enter N/A for 1-4)* | Content *Food, PA, both* | Messages *[info* (food pyramid); *advertising* a product (Coke); take *action (eat more veggies); celebrity role model; OR no signage posted]* | Location *(serving area, cafeteria, wall, eye level, too high/far to read, other; N/A)* |
|---|---|---|---|---|---|---|
| **M1.** Soft drinks, brand name foods, or fundraisers w/ food | ☐ | ☐ | **M1.1** | **M1.2** | **M1.3.** | **M1.4** |
| **M2.** Healthy eating and physical activity (PA) promotion | ☐ | ☐ | **M2.1** | **M2.2** | **M2.3.** | **M2.4** |

**N0. Kitchen/Prep Area. Please rate and answer the following for the KITCHEN/PREPARATION AREAS.**

| Item | Great | Good | Fair | Poor | N/A |
|---|---|---|---|---|---|
| **N1. Is there a kitchen?** | ☐ Yes | | ☐ No *(If no, check "N/A" for N2-N8)* | | |
| **N2. Attractiveness** | ☐ Good condition, bright, clean | | ☐ Poor condition, dark, dirty | | ☐ N/A |
| **N3. Physical structure** *(floors, walls, ceiling)* | ☐ Clean, well kempt, no damage or cracks | ☐ Stained but clean; no repair needed | ☐ Some damage, cracks, peeling paint | ☐ Unkempt, dirty, damaged, peeling paint | ☐ N/A |
| **N4. Kitchen equipment** *(sink, stove/oven, freezers, refrigerators, mixers, etc.)* | ☐ Clean, not damaged | ☐ Stained but clean; not damaged | ☐ Some stains and damage | ☐ Dirty, damaged, repairs needed | ☐ N/A |
| **N5.2. Lighting** | ☐ Bright/adequately lit | | ☐ Dark (*if lights are off, select N/A*) | | ☐ N/A Lights off |
| **N6. Windows present?** | ☐ Yes | | ☐ No | | ☐ N/A |
| **N7. Cleanliness** | ☐ Clean | ☐ Almost clean | ☐ Satisfactory | ☐ Dirty/moldy | ☐ N/A |
| **N8. Clutter** | ☐ No clutter | ☐ Almost no clutter | ☐ Some clutter | ☐ Max. clutter/chaos | ☐ N/A |

**O0. Please rate the following for the SERVING AREA.**

| Item | Great | Good | Fair | Poor | N/A |
|---|---|---|---|---|---|
| **O1. Attractiveness** | ☐ Good condition, bright, clean, child appropriate | | ☐ Poor condition, dark, dirty, *not* child appropriate | | |
| **O2. Physical structure** *Floors, walls, ceiling* | ☐ Clean, well kempt, no damage or cracks | ☐ Stained but clean; no repair needed | ☐ Some damage, cracks, peeling paint | ☐ Unkempt, dirty, damaged, peeling paint | |
| **O3. Equipment condition** *(Counters, display, shelves, etc.)* | ☐ Clean, well kempt, no damage | ☐ Stained but clean; no repair needed | ☐ Some repair or cleaning needed | ☐ Unkempt, dirty, damaged | |
| **O4.2. Lighting** | ☐ Bright/adequately lit | | ☐ Dark (*if lights are off, select N/A*) | | ☐ N/A lights off |
| **O5. Cleanliness** | ☐ Clean | ☐ Almost clean | ☐ Satisfactory | ☐ Dirty/moldy | |
| **O6. Clutter** | ☐ No clutter | ☐ Almost no clutter | ☐ Some clutter | ☐ Max. clutter/chaos | |
| **O7.2. Food attractiveness** | ☐ Fresh, colorful, creatively and cleanly presented | ☐ Most items fresh, colorful, cleanly presented | ☐ Some items fresh, colorful, cleanly presented | ☐ Not fresh, bland colors, unattractive presentation | ☐ N/A no food is present |

**Observer name**: ___________________________________ **State** ______________ **School ID#**________

**O8. Note where the serving area is located:**

**a.** ☐ Inside the cafeteria/eating area
**b.** ☐ A serving window
**c.** ☐ A space separated by at least a door/opening from the cafeteria
**d.** ☐ A separate space, but the fruit/salad bar is in the cafeteria

**P1. Locate the student DAILY menu, displaying today's meal items. Are menu items named:**

**a.** ☐ By item *(e.g., carrots)*
**b.** ☐ Creatively *(e.g., carrot power sticks)*
**c.** ☐ Descriptively *(e.g. freshly picked carrots)*
**d.** ☐ No menu posted/visible (e.g., only small monthly menu calendar)

**P2. If the student menu is in the cafeteria/eating area or serving area, is it visible:**

**a.** ☐ Students preorder meals ahead of time (menu may or may not be posted)
**b.** ☐ Before students are served, along the circulation path (e.g., on the door into the serving area)
**c.** ☐ Only visible once students are in line being served items
**d.** ☐ Posted, but too far away, too high, or too small (e.g., font) to read
**e.** ☐ No menu is posted in the cafeteria or serving area, and there is no preordering

| **Answer the following items about the SERVING AREA:** | | | |
|---|---|---|---|
| **P3. Is one of the 1st three items students see as they enter the serving area a fruit or vegetable?** | ☐ No | ☐ Yes | |
| **P4.1. Students make multiple serving trips (vs. only one time through checkout line allowed).** | ☐ Yes, for all items | ☐ No; only for certain items (*check N/A for P4.2*) | |
| **P4.2. *If yes,* students make multiple serving trips for FV/healthy items only.** | ☐ No | ☐ Yes, for fruits, vegetables, LF white milk | ☐ N/A |
| **P5. Individual food items in the serving area are labeled.** | ☐ No, none | ☐ Yes, some or all | |
| **P7. Are fresh fruits located next to the checkout station?** | ☐ No | ☐ Yes | ☐ N/A no fresh fruit available |
| **P8.1. Is ice cream (or equivalent) available to students?** | ☐ Yes | ☐ No (*check N/A for P8.2*) | |
| **P8.2. *If yes,* the ice cream cooler lid is:** | ☐ Transparent | ☐ Opaque | ☐ N/A |
| **P9.2. Are some/all unhealthy items (chips, cookies, processed foods), located out of reach (e.g., behind counter) or by request only?** | ☐ No | ☐ Yes | ☐ N/A no unhealthy items |
| **P10. Tray rests are available in front of serving stations.** | ☐ No | ☐ Yes | |
| **Q1. Students use serving trays (vs. just plates/bowls).** | ☐ No (*N/A for Q2-3*) | ☐ Yes | |
| **Q2. *If yes*, how many lunch tray <u>colors</u> are available?** | ☐ Only 1 color | ☐ > 1 color | ☐ N/A, no trays |
| **Q3. *If yes*, what is the lunch tray material?** | ☐ Styrofoam, cardboard (bendable) | ☐ Plastic (not bendable) | ☐ N/A, no trays |

**Measure the length and width of each available serving tray *<u>size</u>*. Calculate the area of each tray size in square inches.**

**Q4.1. L** ________ **in** x **W** ________ **in** = ________**in²** **Q6.2. L** ________ **in** x **W** ________ **in** = ________**in²**

**Q5.2. L** ________ **in** x **W** ________ **in** = ________**in²** **Q7.2. L** ________ **in** x **W** ________ **in** = ________**in²**

**Q8. Calculate the average serving tray area by summing the above areas and dividing by the number of tray sizes. Select the largest 2 and smallest 2 sizes if more than four are available:** _______________________________________________

**Q9. Locate utensils available to students. Are plastic forks, spoons, <u>and</u> knives, or all needed utensils for the meal(s) being served available**? ☐ Yes ☐ No

**Q10. Are sporks (combination of spoon and fork) available?** ☐ Yes ☐ No

**Observer name:** ________________________________  **State** ____________  **School ID#**________

**Answer the following questions about fresh fruit:**

**R1.1. Is fresh fruit offered?** ☐ Yes ☐ No (*If no, check N/A* R1.2-4*)

**R1.2 Fresh fruit is served *mostly* from a:** ☐ Metal/plastic tray ☐ Decorative bowl/display ☐ Both ☐ N/A

**R1.3. Fresh fruit is *mostly* served:** ☐ Sliced ☐ Whole ☐ Both ☐ N/A

**How are the <u>majority</u> of the following foods served and presented? Check all that apply, or "N/A" for unavailable items.**

| Item | Individual, pre-measured, or plated servings <u>OR</u> | Self-served from larger trays, bowls, or pots | Wrapped or covered, transparent <u>OR</u> | Wrapped or covered, NOT transparent | N/A |
|---|---|---|---|---|---|
| **R1.4. Fruits & vegetables** | ☐ | ☐ | ☐ | ☐ | ☐ N/A |
| **R2.2. Desserts** | ☐ | ☐ | ☐ | ☐ | ☐ N/A |
| **R3.2. Snack items** | ☐ | ☐ | ☐ | ☐ | ☐ N/A |

**S. Locate the milk cooler/display from which students are served and answer the following questions.**

**S1. What type(s) of milk are offered? Check all that apply.**
**a.** ☐ Only low fat (skim, 1%, 2%), <u>*unflavored*</u> milk
**b.** ☐ Only white, <u>*unflavored*</u> milk is offered.
**c.** ☐ None of the above

**S2. How many rows of different types of milk are displayed as students move through the serving line?**

☐ ONE row of milk (all types of milk are displayed next to each other, in one single row).
*If you select this box, check "N/A" for S3-S4 and answer S5-S6.*

☐ MORE THAN ONE row of milk (milk is displayed in multiple rows, with some types behind the other).
*If you select this box, answer S3-S4 and enter "N/A" for S5-S6.*

**S3. Is the low-fat, unflavored milk located (2+ rows):** ☐ N/A
☐ Behind flavored milk, *not* 1st in line ☐ In front of flavored milk, *not* 1st in line
☐ Behind flavored milk, *1st in line* ☐ In front of flavored milk, *1st in line*

**S4. Is the white, unflavored milk located (2+ rows):** ☐ N/A
☐ Behind flavored milk, *not* 1st in line ☐ In front of flavored milk, *not* 1st in line
☐ Behind flavored milk, *1st in line* ☐ In front of flavored milk, *1st in line*

**S5. Is the low-fat, unflavored milk located (1 row):** ☐ *1st in line* ☐ NOT 1st in line ☐ N/A
**S6. Is the white, unflavored milk located (1 row):** ☐ *1st in line* ☐ NOT 1st in line ☐ N/A

**S7. Count the visible flavored and unflavored milk crates. Is at least 50% of the milk visible to students from the serving line white, unflavored milk?** ☐ Yes ☐ No

**T. If observing during a meal time (or lunch tray photos), observe at least 30 students and add a "tick mark" to the appropriate column as students exit the serving line based on the fruits (F) and vegetables (V) on their tray.**

| ≥1 side fruit | ≥1 side vegetable | Both ≥1 F/V | No F/V | TOTAL |
|---|---|---|---|---|
| | | | | **T1.1** *total students observed=* ________ |
| **T1.2 Total=** | **T1.3 Total=** | **T1.4 Total=** | **T1.5 Total=** | |

**T2. All students had ≥1 side FRUIT on their lunch tray at check out (T1.2+T1.4=T1.1)** ☐ Yes ☐ No

**T3. All students had ≥1 side VEGETABLE on their lunch tray at check out (T1.2+T1.3=T1.1)** ☐ Yes ☐ No

**T4.** List the side fruits and vegetables offered: ________________________________

**T5.** Total number of side fruits and vegetables offered during the meal (salad, applesauce, corn, carrots): ________

**T6. Are at least half (50%) of the items RAW?** ☐ Yes ☐ No

**T7. Are any meal items breaded or fried** (e.g., chicken fingers, mozzarella sticks)? ☐ Yes ☐ No

**Observer name**: ____________________________________ **State** ______________ **School ID#**________

***Pages 9-11 were used for CAFES development and are not required to complete CAFES. Researchers using CAFES to evaluate multiple school cafeterias, however, may find this additional documentation useful.***

**U. Please take photos^ of the following spaces/items:**

☐ (4) ***Kitchen and food preparation spaces/areas***
☐ (4) ***Food/beverage serving spaces/areas***
☐ (All) ***Food/beverage serving <u>stations</u> (see example below)***
☐ (4) ***Cafeteria/eating spaces/areas (see example below)***
☐ ***Photograph a student serving tray next to a ruler (one per tray size and color)***

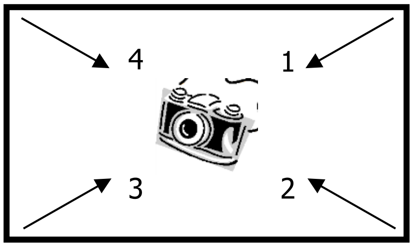


*^ Stand near one corner at a time in each space, face the opposite corner, and take a photo. Repeat for all corners (examples below). The entire room should be captured, and each photo should contain both floor and ceiling.*

*^ If children are present during photography, NO FACES may be photographed.*

***Cafeteria example photos:***

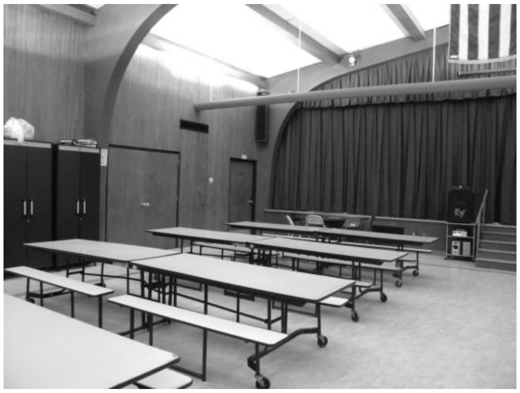
**Corner 1**

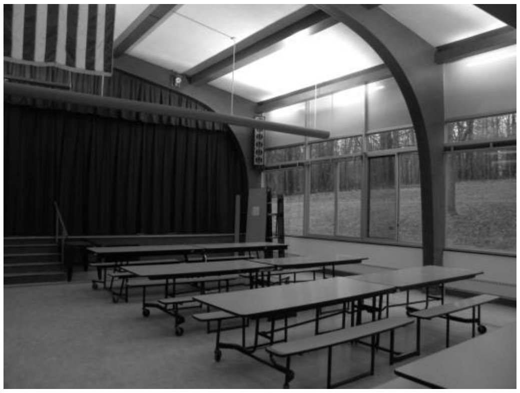
**Corner 2**

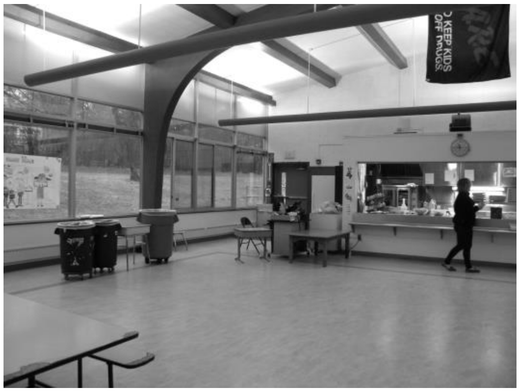
**Corner 3**

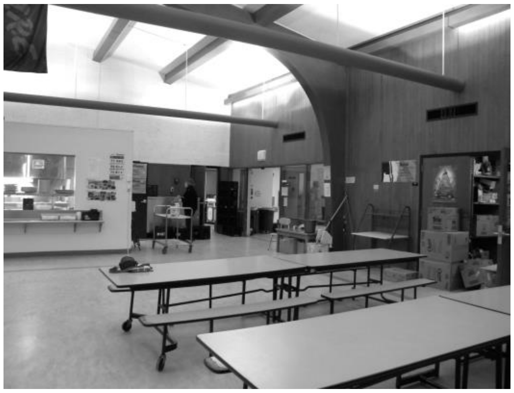
**Corner 4**

***Serving station example photos (be sure to label each item in your sketches on p. 11)***

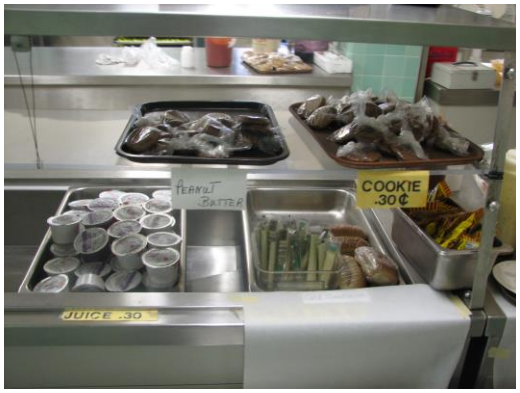


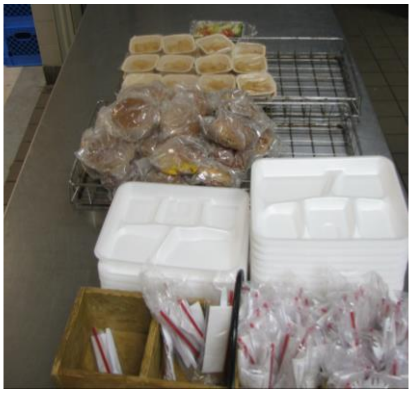

**Observer name**: ___________________________________ **State** ______________ **School ID#**________

**V. Below, please *sketch and label* the food/beverage preparation (kitchen), serving, and eating areas (cafeteria).**

**1. Draw the walls.**
**2. Draw doors/windows.**
**3. Draw the furniture.**

**5. Draw and label:**

- ☐ *Kitchen/prep area*
- ☐ *Serving area*
- ☐ *Menu locations* (**M**)
- ☐ *Storage areas*
- ☐ *Student entry/exit*
- ☐ *Number all food/beverage locations* (**#**)
- ☐ *Payment location*
- ☐ *Student circulation paths: entry, seating, food/bev, payment, trash, exit*
- ☐ *Disposal/trash area*
- ☐ *Tables and seating* (draw exact number of tables, note # chairs, bench)
- ☐ *Healthy food and PA signage* (**S**)

**Example sketch:**

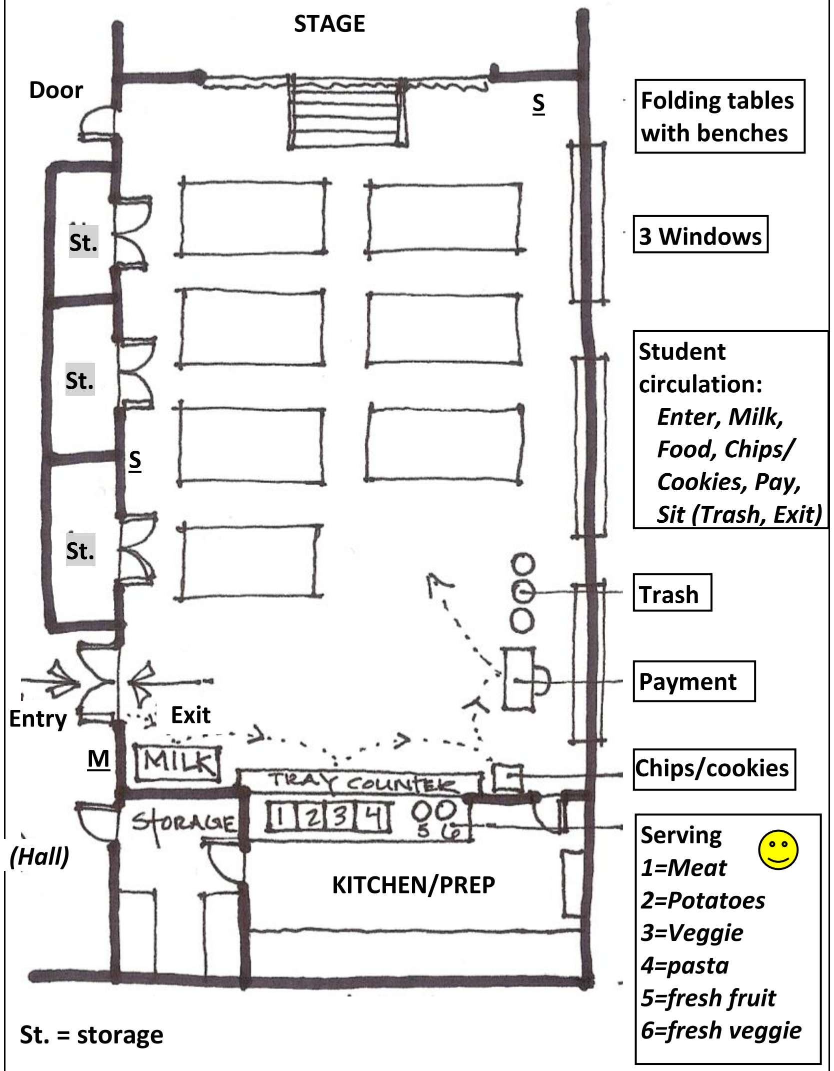


**St. = storage**

Observer name: ___________________________________ State ______________ School ID#________

**W. Below, please photograph, sketch, AND <u>label</u> *each* food and beverage serving location according to the instructions.**

**Example sketch: #n/a. MILK refrigerator**

*Front of stainless steel refrigerator*

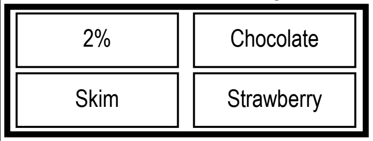


*View after student lifts opaque top; beverage label is printed on cardboard cartons; cartons are all the same except for label color/text*

**Example sketch: #1-6. Warm food serving station; fruit bowls**

*Front*

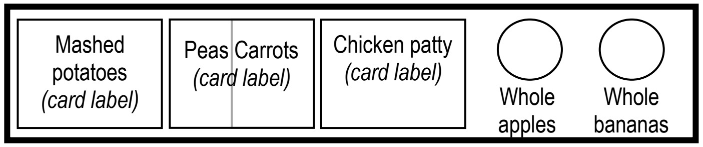


*Metal trays, uncovered, not behind glass on metal counter top; food items labeled cover descriptively on white card with black handwritten text*

*Opaque red glass bowls on metal labeled counter, no item label*

**INSTRUCTIONS**:

Carefully *photograph* each food and beverage serving location numbered on your previous sketch.

*Sketch* each food and beverage serving location numbered on your previous sketch:

**A. List # from previous sketch**

**B. Title the station**

**C. Draw the station**

**D**. Label individual items

**E. Are individual items labeled?** If yes, describe item labels.

**F**. Describe how each item is served:

***- Container type***
*e.g. tray, bowl, basket, pot*

***- Container material***
*e.g. metal, glass, plastic*

*-Is container* ***transparent or opaque?***

-Is the container ***covered*** *(i.e., students have to remove before serving)?*

-Is the ***cover opaque*** or ***transparent***?

**- Are students served or serve themselves?**

**- Are items pre-portioned?**

**-Describe surfaces below serving containers and materials** *e.g., Metal or glass counter, plastic/metal*

**Observer name**: ___________________________________ **State** ______________ **School ID#**________

**Additional Notes** *(contact info, intervention suggestions, relevant policies and programs e.g., recess before lunch, fruit and vegetable snack or backpack program, etc.):*

______________________________________________________________________________________________________

______________________________________________________________________________________________________

______________________________________________________________________________________________________

______________________________________________________________________________________________________

______________________________________________________________________________________________________

______________________________________________________________________________________________________

______________________________________________________________________________________________________

______________________________________________________________________________________________________

______________________________________________________________________________________________________

______________________________________________________________________________________________________

______________________________________________________________________________________________________

______________________________________________________________________________________________________

______________________________________________________________________________________________________

______________________________________________________________________________________________________

______________________________________________________________________________________________________

______________________________________________________________________________________________________

______________________________________________________________________________________________________

______________________________________________________________________________________________________

______________________________________________________________________________________________________

## CAFETERIA ASSESSMENT FOR ELEMENTARY SCHOOLS (CAFES) – CODED VERSION                    School ID#: _____

**INSTRUCTIONS: The CAFES tool evaluates 2nd-5th grade elementary school cafeterias and students. Please complete the following items as noted. Green text indicates scoring information that accompanies the scoring spreadsheet file.**

**= when possible, complete item *just before/during* a meal time**          **= DO NOT complete item during a meal time**

**SUPPLY LIST:** *CAFES forms, Camera (batteries/memory cards), Ruler & tape measure, writing utensils & extra paper*

***A (*) indicates an "N/A" item that should NOT be included in the total CAFES item count if selected/checked.***

**A0. Please enter the following information about the CAFES tool user and the observed school.**

**A1-2. CAFES user name**: ___________________________________ **A3. School name**: _____________________________________

**A4. School street address/city:** _____________________________________________ **A5. State:** ________ **A6. Zip:** ___________

**A7. Participating Grades** (circle all that apply): 2nd 3rd 4th 5th      **A8. CAFES Observation Date:** _____ / _______ / _______

**A9. Is this observation being completed during a lunch period?** ☐ Yes ☐ No

**A10. Is this observation being completed while lunch food is present in the serving area?** ☐ Yes ☐ No

**B0. Please ask Food Service staff and/or the Principal the following questions.**

**B1. How many cafeterias/eating areas are used for student lunch?** *If more than one cafeteria or eating area is used, evaluate the space used by the participating grades, or complete separate CAFES evaluations for each space.* ___________

**B2. Select the grade levels that use the eating area being evaluated by CAFES:** ☐ 2nd ☐ 3rd ☐ 4th ☐ 5th

| No. | Question: | Yes | No | Additional response |
|---|---|---|---|---|
| B3.1 | **Are food or beverage vending machines available for student use during lunch?** *If no, select N/A for B3.1b.* | ☐ 0 | ☐ 1 | **B3.1a** # Machines:______ |
| B3.1b* | ***If yes,* are only fruits and vegetables available?** | ☐ 1 | ☐ 0 | ☐ N/A (*) |
| B3.2 | **Do fundraisers involving food occur DURING LUNCH TIME?** | ☐ 0 | ☐ 1 | |
| B3.3 | **Do fundraisers involving food occur IN THE CAFETERIA OR EATING AREA(S) during lunch time?** | ☐ 0 | ☐ 1 | |
| B3.4* | **Are different portion sizes available for students of different ages/grades (K–3rd / 4th-6th / 7th+)?** | ☐ 1 | ☐ 0 | |
| B3.5 | **Are school lunches prepared by a contracted/outside food company?** | ☐ | ☐ | |
| B3.6 | **What is the total student population?** ____________________ *students* | | | **B3.10 Length of each period:** _____________________ *minutes* |
| B3.7 | **How many students eat per LUNCH period?** _______________ *students/period* | | | |
| B3.8 | **How many lunch periods occur per day?** __________________ *periods* | | | |
| B3.9 | **Note the start and end times of each lunch period**: _____________________________________________________ | | | |

**C0. Please ask Food Service staff the following questions.**

**C1. Are meals prepared (not just reheated or assembled) at the school?** ☐ Yes (1) ☐ No (0)

**C2. Considering the last year, which of the following factors HELPED your school cafeteria's ability to provide healthier food choices? Select all that apply.** (a & b: 0=<u>un</u>check, 1=check; c: 0= check)

**a.** ☐ Suitable equipment available (1)   **b.** ☐ Adequate storage space available (1)   **c.** ☐ Neither (0)

**C3A. Considering the last year, which of the following factors HELPED your school cafeteria's ability to provide healthier food choices? Select all that apply.** (a & b: 0=<u>un</u>check, 1=check; c: 0= check)

**a.** ☐ Suitable preparation area available (1) **b.** ☐ Adequate display space available (1)   **c.** ☐ Neither (0)

**C3B. Considering the last year, did a lack of storage space LIMIT your school cafeteria's ability to provide healthier food choices?** ☐ Yes (0) ☐ No (1)

**Observer name:** ______________________________ **State** _________ **School ID#**________________

**C4. Students serve themselves for (check all that apply):**

**a.** ☐ A la carte lunch entrees (0=check, 1=uncheck) **b.** ☐ A la carte lunch sides (0=check, 1=uncheck) **c.** ☐ Salad/veggies/fruit (0=uncheck, 1=check) **d.** ☐ None of the above (0=check, 1=uncheck)

**C5. Is the "offer-versus-serve" option used during lunch (students are asked whether or not they want a particular meal item) for any of the following items? Check all that apply.**

**a.** ☐ A la carte lunch entrees (0=check, 1=uncheck) **b.** ☐ A la carte lunch sides (0=check, 1=uncheck) **c.** ☐ Salad/veggies/fruit (0=uncheck, 1=check) **d.** ☐ None of the above (0=check, 1=uncheck)

| **D0.** ***Please ask Food Service staff how often each of the following items are offered during lunch, on average, each week and check the average number of days per 5-day week.*** | | **Average days per week** | | | | | |
|---|---|---|---|---|---|---|---|
| | | **0** | **1** | **2** | **3** | **4** | **5** |
| **D1** | Food reheated for students' lunches | 6 | 4 | 3 | 2 | 1 | 0 |
| **D2** | TWO or more different entrees/main courses | 0 | 1 | 2 | 3 | 4 | 6 |
| **D3** | TWO or more different vegetables | 0 | 1 | 2 | 3 | 4 | 6 |
| **D4** | TWO or more different fruits | 0 | 1 | 2 | 3 | 4 | 6 |
| **D5.1** | Salad | 0 | 1 | 2 | 3 | 4 | 6 |
| **D5.2** | *Note if salad is an entrée, side, or both (or n/a)*: ______________________ | | | | | | |
| **D6** | Whole grains | 0 | 1 | 2 | 3 | 4 | 6 |
| **D7** | Pizza | 6 | 4 | 3 | 2 | 1 | 0 |
| **D8** | French fried or *breaded* potatoes, hash browns, tater tots, including baked/reheated | 6 | 4 | 3 | 2 | 1 | 0 |
| **D9** | Spaghetti, macaroni, or other pasta | 6 | 4 | 3 | 2 | 1 | 0 |
| **D10** | Cookies, crackers, chips, pastries, cakes, other baked goods ***not*** low in fat | 6 | 4 | 3 | 2 | 1 | 0 |
| **D11** | Low fat or fat free cookies, crackers, chips, pastries, cakes, other baked goods | 6 | 4 | 3 | 2 | 1 | 0 |
| **D12** | Ice cream or frozen yogurt ***not*** low in fat | 6 | 4 | 3 | 2 | 1 | 0 |
| **D13** | Low fat or fat free ice cream, frozen yogurt, sherbet, slushy, icy | 6 | 4 | 3 | 2 | 1 | 0 |
| **D14** | A la carte lunch | 6 | 4 | 3 | 2 | 1 | 0 |

**E0. Please ask Food Service staff what beverages are available to students during lunch.**

**E1. What type of milk is available for students to purchase during lunch? Check all that apply.**

**a.** ☐ Whole/full-fat UNFLAVORED milk and/or soy milk (0 if checked, 1 if unchecked)

**b.** ☐ Whole/full-fat FLAVORED milk and/or soy milk (0 if checked, 1 if unchecked)

**c.** ☐ Reduced/low-fat, skim, or fat-free UNFLAVORED white and/or soy milk (1 if checked, 0 if unchecked)

**d.** ☐ Reduced/low-fat, skim, or fat-free FLAVORED milk and/or soy milk (0 if checked, 1 if unchecked)

**e.** ☐ ONLY reduced/low-fat, skim, or fat-free milk available (including white, flavored, and soy milks) (1 if checked, 0 if *unchecked*)

**f.** ☐ ONLY UNFLAVORED reduced/low-fat, skim, or fat-free milk available (including white and soy milks) (1 if checked, 0 if *unchecked*)

**g.** ☐ No milk is available for students to purchase during lunch (0 if checked)

**E2. Are juice and other sweetened beverages available to students during lunch?**

**a.** ☐ Yes, but only 100% juice (2) **b.** ☐ Yes, <100% juice and other sweetened beverages (0) **c.** ☐ No (3)

**E3. Is water available to students during lunch (not just a drinking fountain without cups)?** ☐ Yes (1) ☐ No (0)

**Observer name:** ______________________________ **State** _________ **School ID#**________________

## F0. Cafeteria Ambient Environment

**Please rate the following for the CAFETERIA/EATING AREA.** Remember to complete separate CAFES evaluations if there are multiple cafeterias/eating areas.

| | Great (1) | Good (1) | Fair (0) | Poor (0) | Other |
|---|---|---|---|---|---|
| **F1. TEMPERATURE** | ☐ OK | | ☐ Hot or cold | | |
| **F2. Is AIR CONDITIONING available?** | ☐ Yes | | ☐ No A/C | | |
| **F3.2. LIGHTING** | ☐ Bright/Adequately lit | | ☐ Dark (*if lights are off, select N/A*) | | ☐ N/A lights off (*) *F3.1* |
| **F4.2. ODOR** | ☐ No smell or pleasant smell | ☐ Noticeable but not unpleasant odor | ☐ Slightly unpleasant odor | ☐ Strong, unpleasant odor | ☐ Food is present during observation of odor *F4.1* |
| **F5.2. NOISE** | ☐ Very quiet | ☐ Soft voices | ☐ Loud talking | ☐ Yelling/screaming | ☐ N/A no students (*) *F5.1* |
| **F6. MUSIC during lunch?** | ☐ Yes | | ☐ No | | |

## G0. Cafeteria Appearance

**Please rate the following for the CAFETERIA/EATING AREA** (complete separate CAFES forms for each area)**.**

| | Great (1) | Good (1) | Fair (0) | Poor (0) | Other (*) |
|---|---|---|---|---|---|
| **G1. Attractiveness** | ☐ Good physical condition, bright, clean, child appropriate | | ☐ Poor physical condition, dark, dirty, not child appropriate | | |
| **G2. Physical structure** *(floors, walls, ceiling)* | ☐ Clean, well kempt, no damage or cracks | ☐ Stained but clean; no repair needed | ☐ Some damage, cracks, peeling paint | ☐ Unkempt, dirty, peeling paint, leaks, damaged | |
| **G3. Clutter** | ☐ No clutter | ☐ Almost no clutter | ☐ Some clutter | ☐ Max. clutter/chaos | |
| **G4. Cleanliness** | ☐ Clean | ☐ Almost clean | ☐ Satisfactory | ☐ Dirty/moldy | |
| **G5.2. Condition of tables and seating** | ☐ Clean, no damage | ☐ Stained but clean; no damage | ☐ Stains, needs some repairs and paint | ☐ Dirty, damaged, paint needed | ☐ (*) *G5.1* Not present |
| **G6.2. Attractiveness of tables & seating** | ☐ Good physical condition, bright, clean, child appropriate | | ☐ Poor physical condition, dark, dirty, not child appropriate | | ☐ (*) *G6.1* Not present |

## H. Cafeteria Layout

**Are any of the following items visible from the cafeteria/eating area?**

**H1. Food or beverages** ☐ (1) Yes, all ☐ (1) No, all in separate room ☐ (1) Only fruit, vegetables, whole grains, skim white milk ☐ (0) Only other items

**H2. Food vending machines** ☐ Yes (0) ☐ No (1)

**H3. Beverage vending machines** ☐ Yes (0) ☐ No (1)

**H4. Observe student circulation (path from the entrance to serving area, seating, trash, and exit) during lunch, or ask food service staff if the student circulation is:**

☐ (0) Unclear: lots of overlapping paths and areas for congestion

☐ (1) Clear: no/only minor overlapping: enter, food, seating, trash, exit without interfering paths or chaos

**H5. Are there any obstructions in the cafeteria/eating or serving areas that affect student movement (e.g., columns, piers, pipes)?** ☐ Yes (0) ☐ No (1)

***A (*) indicates an "N/A" item that should NOT be included in the total CAFES item count if selected/checked.***

**Observer name**: ________________________________ **State** _________ **School ID#**________________

**I. Please follow the instructions to calculate the area of the cafeteria/eating area.** If your school has more than one cafeteria/eating area for elementary students, complete separate CAFES forms for each space.

**I1. Using the space below, please draw the shape of the cafeteria/eating area floor (e.g., square, rectangle, L-shaped, etc.). Measure the length of each cafeteria wall in feet. On the drawing, label each wall with the appropriate measurement. Please convert all measurements to feet, up to two decimal places (9 ft - 3 in = 9.25 ft).**

*I1A. If your school is participating in a CAFES research study, please take a picture of the drawing you just created and share it with the researchers.*

**I2. Calculate the total cafeteria floor area in square feet (SF), up to two decimal places, and enter the result.**
For example, a 20' x 10'-4" (10.33) rectangular space has an area of 206.67 SF. ___________________________________
*(area formulas: square/rectangle = Length x Width; triangle = ½ Base x Height; circle = 3.14 x radius$^2$) The area of a triangular space is calculated using the formula 1/2(base x height) where the base and height are entered in square feet (SF), up to two decimal places. The area of a circular space is 3.14 x radius x radius.* Enter this answer above, and on the last page for K9 scoring.

**I3. Measure the highest and lowest ceiling heights** *(feet, inches)*. If ceiling heights are too high to measure, measure one cinder block, brick, or wall tile and count those, or estimate the height. Convert measurements to feet, up to two decimal places (e.g., enter 9.5 for a 9'-6" ceiling; enter 9.25 for a 9'-3" ceiling), and enter here:

**I3.1. Ceiling height, high:** ____________________ **FT** **I3.2. Ceiling height, low:** ____________________ **FT**

**I4. Calculate the total cafeteria wall area in feet, up to two decimal places. For each wall, multiply its length and height to calculate its area, then sum the areas of all walls. Enter the sum here:** ______________________________________ **FT**

**I5**. If I2 cafeteria floor area is <u><</u>3,000 SF: I3.1 *High* ceiling height <12'-0 = **Enter 0**; OR I3.1 *High* ceiling height <u>></u>12'-0" **Enter 1**
If I2 cafeteria floor area is >3,000 SF: I3.1 *High* ceiling height <14'-0 = **Enter 0**; OR I3.1 *High* ceiling height <u>></u>14'-0" **Enter 1**

**J0. Please follow the instructions to calculate the area of windows present in the cafeteria/eating area.**

**J1. Does the cafeteria/eating area have windows?** ☐ **Yes** *(continue to J2)* ☐ **No** *(skip to section K.)*

**J2. Cafeteria Window Area. Measure each window height** *(h)* **and width** *(w)* **in feet (two decimal places).** Measure the window glass from top to bottom (h) and left to right (w). Ignore any divisions in the glass within a single window.

| **Size** | ***Height*** (ft) x | **Width** (ft) x | **Quantity** = | **Area Total** | **Size** | ***Height*** (ft) x | **Width** (ft) x | **Quantity** = | **Area Total** |
|---|---|---|---|---|---|---|---|---|---|
| 1 | _________ | _________ | _________ | __________ | 7 | _________ | _________ | _________ | __________ |
| 2 | _________ | _________ | _________ | __________ | 8 | _________ | _________ | _________ | __________ |
| 3 | _________ | _________ | _________ | __________ | 9 | _________ | _________ | _________ | __________ |
| 4 | _________ | _________ | _________ | __________ | 10 | _________ | _________ | _________ | __________ |
| 5 | _________ | _________ | _________ | __________ | 11 | _________ | _________ | _________ | __________ |
| 6 | _________ | _________ | _________ | __________ | 12 | _________ | _________ | _________ | __________ |

**J3. Total window area**: For each window type, calculate the total area in SF (H x W X Quantity), then sum all window areas and enter the sum here in square feet, up to two decimal places.: ___________________________________________

**J4. Calculate the % of cafeteria walls that are windows:** J3 Total window area / I4 total wall area = ______________________
Enter **0** if the percentage is <10% (0.10) or no windows Enter **1** if the percentage is <u>></u> 10% (0.25)

Observer name: ______________________________________ State _______________ School ID#________

## K0. Cafeteria Furniture

**K1. The following questions ask about the tables and seating in the student cafeteria/eating area.**

| | |
|---|---|
| **K2.1 How many tables are RECTANGULAR with ATTACHED seating?** | # |
| **K2.2 How many tables are RECTANGULAR with DETACHED chairs?** | # |
| **K2.3 How many tables are CIRCULAR/SQUARE with ATTACHED seating?** | # |
| **K2.4 How many tables are CIRCULAR/SQUARE with DETACHED chairs?** | # |
| **K3.1 Enter the total number of attached individual seats:** | # |
| **K3.2 Enter the total number of detached individual seats:** | # |
| **K3.3 Enter the total number of attached benches:** | # |

| | | | |
|---|---|---|---|
| **K4. Do students sit on individual seats or benches?** | ☐ Benches (0) | ☐ Individual Seats (1) | ☐ Both (1) |
| **K5. Are seats attached to the tables?** | ☐ Yes (0) | ☐ No (1) | ☐ Both (1) |
| **K6. Is there a "sharing" table where students can leave or take uneaten food items?** | ☐ Yes (1) | ☐ No (0) | |
| **K7. Table shapes are:** | ☐ All rectangular (0) | ☐ Some or all are circular/square (1) | |

**K8. Crowding Table:** Total # students (**B3.6**) / # meal periods (**B3.8**) / # tables (**K2.1+K2.2+K2.3+K2.4**) = ____________________

☐ Enter **0 if answer is >10 students per table** ☐ Enter **1 if answer is ≤10 students per table**

**K9. See last page for K9 coding and scoring instructions.**

## Cafeteria Windows

**L0. Please complete the following items about cafeteria/eating area WINDOWS on exterior walls.**

| Item | Response (1) | Response (0) | Response (0) | N/A* |
|---|---|---|---|---|
| **L1. Does the cafeteria/eating area have WINDOWS/SKYLIGHTS?** | ☐ Yes (includes skylights) | ☐ Interior windows only | ☐ No *(check N/A for L2-L7)* | |
| **L2. Rate the condition of the windows** *(not skylights).* | ☐ Clean, not broken or damaged, transparent; only minor cleaning, painting, or repairs needed | ☐ Dirty, cracked, damaged, or broken; peeling paint; not transparent | ☐ No windows/only skylights | ☐ N/A* |
| **L3. How natural *(trees, grass, water)* is the view from the windows?** | ☐ More than ½ natural | ☐ Less than ½ natural | ☐ No view/only non-natural interior views | ☐ N/A* |
| **L4. Are the windows operable?** | ☐ All, some | ☐ None/interior windows only | | ☐ N/A* |
| **L5. Are the windows transparent** *(i.e., not tinted, opaque, or too dirty to see through)?* | ☐ All, some | ☐ None are transparent | | ☐ N/A* |
| **L6. Do windows have treatments such as blinds, curtains, or shades to control light?** | ☐ All, some | ☐ No treatments | | ☐ N/A* |
| **L7. Do the windows have screens?** | ☐ All, some | ☐ No screens/interior windows only | | ☐ N/A* |

***A (*) indicates an "N/A" item that should NOT be included in the total CAFES item count if selected/checked.***

Observer name: ______________________________ State ____________ School ID#________

## M0. Advertisements – Cafeteria and serving areas

**Inside the CAFETERIA/EATING and SERVING AREA, are advertisements, signs, and/or flyers visible for:**

| Topic | Yes | No | Quantity # (If no, enter N/A for 1-4) | Content *Food, PA, both* | Messages *[info* (food pyramid); *advertising* a product (Coke); take *action (eat more veggies); celebrity role model; OR no signage posted]* | Location *(serving area, cafeteria, wall, eye level, too high/far to read, other; N/A)* |
|---|---|---|---|---|---|---|
| **M1.** Soft drinks, brand name foods, or fundraisers w/ food | 0 ☐ | 1 ☐ | **M1.1** | **M1.2** | **M1.3.** | **M1.4** |
| **M2.** Healthy eating and physical activity (PA) promotion | 1 ☐ | 0 ☐ | **M2.1** | **M2.2** | **M2.3.** | **M2.4** |

## N0. Kitchen/Prep Area. Please rate and answer the following for the KITCHEN/PREPARATION AREAS.

| Item | Great (1) | Good (1) | Fair (0) | Poor (0) | N/A (*) |
|---|---|---|---|---|---|
| **N1. Is there a kitchen?** | ☐ Yes | | ☐ No *(If no, check "N/A" for N2-N8)* | | |
| **N2. Attractiveness** | ☐ Good condition, bright, clean | | ☐ Poor condition, dark, dirty | | ☐ N/A* |
| **N3. Physical structure** *(floors, walls, ceiling)* | ☐ Clean, well kempt, no damage or cracks | ☐ Stained but clean; no repair needed | ☐ Some damage, cracks, peeling paint | ☐ Unkempt, dirty, damaged, peeling paint | ☐ N/A* |
| **N4. Kitchen equipment** *(sink, stove/oven, freezers, refrigerators, mixers, etc.)* | ☐ Clean, not damaged | ☐ Stained but clean; not damaged | ☐ Some stains and damage | ☐ Dirty, damaged, repairs needed | ☐ N/A* |
| **N5.2. Lighting** | ☐ Bright/adequately lit | | ☐ Dark (*if lights are off, select N/A*) | **N5.1:** | ☐ N/A Lights off * |
| **N6. Windows present?** | ☐ Yes | | ☐ No | | ☐ N/A* |
| **N7. Cleanliness** | ☐ Clean | ☐ Almost clean | ☐ Satisfactory | ☐ Dirty/moldy | ☐ N/A* |
| **N8. Clutter** | ☐ No clutter | ☐ Almost no clutter | ☐ Some clutter | ☐ Max. clutter/chaos | ☐ N/A* |

## O0. Please rate the following for the SERVING AREA.

| Item | Great (1) | Good (1) | Fair (0) | Poor (0) | N/A (*) |
|---|---|---|---|---|---|
| **O1. Attractiveness** | ☐ Good condition, bright, clean, child appropriate | | ☐ Poor condition, dark, dirty, *not* child appropriate | | |
| **O2. Physical structure** *Floors, walls, ceiling* | ☐ Clean, well kempt, no damage or cracks | ☐ Stained but clean; no repair needed | ☐ Some damage, cracks, peeling paint | ☐ Unkempt, dirty, damaged, peeling paint | |
| **O3. Equipment condition** *(Counters, display, shelves, etc.)* | ☐ Clean, well kempt, no damage | ☐ Stained but clean; no repair needed | ☐ Some repair or cleaning needed | ☐ Unkempt, dirty, damaged | |
| **O4.2. Lighting** | ☐ Bright/adequately lit | | ☐ Dark (*if lights are off, select N/A*) | **O4.1:** | ☐ N/A lights off (*) |
| **O5. Cleanliness** | ☐ Clean | ☐ Almost clean | ☐ Satisfactory | ☐ Dirty/moldy | |
| **O6. Clutter** | ☐ No clutter | ☐ Almost no clutter | ☐ Some clutter | ☐ Max. clutter/chaos | |
| **O7.2. Food attractiveness** | ☐ Fresh, colorful, creatively and cleanly presented | ☐ Most items fresh, colorful, cleanly presented | ☐ Some items fresh, colorful, cleanly presented | ☐ Not fresh, bland colors, unattractive presentation | ☐ N/A no food is present (*) O7.1 |

***A (*) indicates an "N/A" item that should NOT be included in the total CAFES item count if selected/checked***

**Observer name:** ____________________________________ **State** ______________ **School ID#**________

**O8. Note where the serving area is located:**

**a.** ☐ Inside the cafeteria/eating area
**b.** ☐ A serving window
**c.** ☐ A space separated by at least a door/opening from the cafeteria
**d.** ☐ A separate space, but the fruit/salad bar is in the cafeteria

**P1. Locate the student DAILY menu, displaying today's meal items. Are menu items named:**

**a.** ☐ (0) By item *(e.g., carrots)*
**b.** ☐ (1) Creatively *(e.g., carrot power sticks)*
**c.** ☐ (1) Descriptively *(e.g. freshly picked carrots)*
**d.** ☐ (0) No menu posted/visible (e.g., only small monthly menu calendar)

**P2. If the student menu is in the cafeteria/eating area or serving area, is it visible:**

**a.** ☐ (1) Students preorder meals ahead of time (menu may or may not be posted)
**b.** ☐ (1) Before students are served, along the circulation path (e.g., on the door into the serving area)
**c.** ☐ (0) Only visible once students are in line being served items
**d.** ☐ (0) Posted, but too far away, too high, or too small (e.g., font) to read
**e.** ☐ (0) No menu is posted in the cafeteria or serving area, and there is no preordering

| **Answer the following items about the SERVING AREA:** | **(0)** | **(1)** | **N/A*** (no score) |
|---|---|---|---|
| **P3. Is one of the 1st three items students see as they enter the serving area a fruit or vegetable?** | ☐ No | ☐ Yes | |
| **P4.1. Students make multiple serving trips (vs. only one time through checkout line allowed).** | ☐ Yes, for all items | ☐ No; only for certain items (*check N/A for P4.2*) | |
| **P4.2. *If yes,* students make multiple serving trips for FV/healthy items only.** | ☐ No | ☐ Yes, for fruits, vegetables, LF white milk | ☐ N/A* P6 |
| **P5. Individual food items in the serving area are labeled.** | ☐ No, none | ☐ Yes, some or all | |
| **P7. Are fresh fruits located next to the checkout station?** | ☐ No | ☐ Yes | ☐ N/A no fresh fruit available* |
| **P8.1. Is ice cream (or equivalent) available to students?** | ☐ Yes | ☐ No (*check N/A for P8.2*) | |
| **P8.2. *If yes,* the ice cream cooler lid is:** | ☐ Transparent | ☐ Opaque | ☐ N/A* |
| **P9.2. Are some/all unhealthy items (chips, cookies, processed foods), located out of reach (e.g., behind counter) or by request only?** | ☐ No | ☐ Yes | ☐ N/A no (***9.1**) unhealthy items |
| **P10. Tray rests are available in front of serving stations.** | ☐ No | ☐ Yes | |
| **Q1. Students use serving trays (vs. just plates/bowls).** | ☐ No (*N/A for Q2-3*) | ☐ Yes | |
| **Q2. *If yes*, how many lunch tray <u>colors</u> are available?** | ☐ Only 1 color | ☐ > 1 color | ☐ N/A, no trays* |
| **Q3. *If yes*, what is the lunch tray material?** | ☐ Styrofoam, cardboard (bendable) | ☐ Plastic (not bendable) | ☐ N/A, no trays* |

**Measure the length and width of each available serving tray *<u>size</u>*. Calculate the area of each tray size in square inches.**

**Q4.1. L** ________ **in** x **W** ________ **in** = ________$in^2$ **Q6.2. L** ________ **in** x **W** ________ **in** = ________$in^2$

**Q5.2. L** ________ **in** x **W** ________ **in** = ________$in^2$ **Q7.2. L** ________ **in** x **W** ________ **in** = ________$in^2$

**Q8. Calculate the average serving tray area by summing the above areas and dividing by the number of tray sizes. Select the largest 2 and smallest 2 sizes if more than four are available:** ______________________________________________

Enter **0** if the area is $\leq$ 100 $in^2$ Enter **1** if the area is > 100 in2 (N/A* if no trays are used)

**Q9. Locate utensils available to students. Are plastic forks, spoons, <u>and</u> knives, or all needed utensils for the meal(s) being served available**? ☐ Yes (1) ☐ No (0)

**Q10. Are sporks (combination of spoon and fork) available?** ☐ Yes ☐ No (no coding)

***A (*) indicates an "N/A" item that should NOT be included in the total CAFES item count if selected/checked.***

Observer name: ______________________________ State ______________ School ID#________

Answer the following questions about fresh fruit:

**R1.1. Is fresh fruit offered?** ☐ Yes (1) ☐ No (0) (*If no, check N/A* R1.2-4*)

**R1.2 Fresh fruit is served *mostly* from a:** ☐ Metal/plastic tray (0) ☐ Decorative bowl/display (1) ☐ Both (1) ☐ N/A (*)

**R1.3. Fresh fruit is *mostly* served:** ☐ Sliced (1) ☐ Whole (0) ☐ Both (1) ☐ N/A (*)

**How are the majority of the following foods served and presented? Check all that apply, or "N/A" for unavailable items.**

| Item | Individual, pre-measured, or plated servings OR | Self-served from larger trays, bowls, or pots | Wrapped or covered, transparent OR | Wrapped or covered, NOT transparent | N/A |
|---|---|---|---|---|---|
| R1.4. Fruits & vegetables | ☐ 0 | ☐ 1 | ☐ 1 | ☐ 0 | ☐ N/A* |
| R2.2. Desserts | ☐ 1 | ☐ 0 | ☐ 0 | ☐ 1 | ☐ N/A* R2.1 |
| R3.2. Snack items | ☐ 1 | ☐ 0 | ☐ 0 | ☐ 1 | ☐ N/A* R3.1 |

**S. Locate the milk cooler/display from which students are served and answer the following questions.**

**S1. What type(s) of milk are offered? Check all that apply.**
**(1 if checked, 0 if unchecked for each of the 1st two options)**

a. ☐ Only low fat (skim, 1%, 2%), *unflavored* milk
b. ☐ Only white, *unflavored* milk is offered.
c. ☐ None of the above (0 if checked)

**S2. How many rows of different types of milk are displayed as students move through the serving line?**

☐ ONE row of milk (all types of milk are displayed next to each other, in one single row).
*If you select this box, check "N/A" for S3-S4 and answer S5-S6.*

☐ MORE THAN ONE row of milk (milk is displayed in multiple rows, with some types behind the other).
*If you select this box, answer S3-S4 and enter "N/A" for S5-S6.*

**S3. Is the low-fat, unflavored milk located (2+ rows):** ☐ N/A*

☐ (0) Behind flavored milk, *not* 1st in line ☐ (1) In front of flavored milk, *not* 1st in line
☐ (0) Behind flavored milk, *1st in line* ☐ (1) In front of flavored milk, *1st in line*

**S4. Is the white, unflavored milk located (2+ rows):** ☐ N/A*

☐ (0) Behind flavored milk, *not* 1st in line ☐ (1) In front of flavored milk, *not* 1st in line
☐ (0) Behind flavored milk, *1st in line* ☐ (1) In front of flavored milk, *1st in line*

**S5. Is the low-fat, unflavored milk located (1 row):** ☐ (1) *1st in line* ☐ (0) NOT 1st in line ☐ N/A*

**S6. Is the white, unflavored milk located (1 row):** ☐ (1) *1st in line* ☐ (0) NOT 1st in line ☐ N/A*

**S7. Count the visible flavored and unflavored milk crates. Is at least 50% of the milk visible to students from the serving line white, unflavored milk?** ☐ Yes (1) ☐ No (0)

**T. If observing during a meal time (or lunch tray photos), observe at least 30 students and add a "tick mark" to the appropriate column as students exit the serving line based on the fruits (F) and vegetables (V) on their tray.**

| $\geq$1 side fruit | $\geq$1 side vegetable | Both $\geq$1 F/V | No F/V | TOTAL |
|---|---|---|---|---|
| | | | | **T1.1** *total students observed=* ________ |
| **T1.2 Total=** | **T1.3 Total=** | **T1.4 Total=** | **T1.5 Total=** | |

**T2. All students had $\geq$1 side FRUIT on their lunch tray at check out (T1.2+T1.4=T1.1)** ☐ Yes (1) ☐ No (0)

**T3. All students had $\geq$1 side VEGETABLE on their lunch tray at check out (T1.2+T1.3=T1.1)** ☐ Yes (1) ☐ No (0)

**T4.** List the side fruits and vegetables offered: ______________________________

**T5.** Total number of side fruits and vegetables offered during the meal (salad, applesauce, corn, carrots): __________

**T6. Are at least half (50%) of the items RAW?** ☐ Yes (1) ☐ No (0)

**T7. Are any meal items breaded or fried** (e.g., chicken fingers, mozzarella sticks)? ☐ Yes (0) ☐ No (1)

Observer name: ______________________________________ State _______________ School ID#_________

***Pages 9-11 were used for CAFES development and are not required to complete CAFES. Researchers using CAFES to evaluate multiple school cafeterias, however, may find this additional documentation useful.***

**U. Please take photos^ of the following spaces/items:**

- ☐ (4) ***Kitchen and food preparation spaces/areas***
- ☐ (4) ***Food/beverage serving spaces/areas***
- ☐ (All) ***Food/beverage serving stations (see example below)***
- ☐ (4) ***Cafeteria/eating spaces/areas (see example below)***
- ☐ ***Photograph a student serving tray next to a ruler (one per tray size and color)***

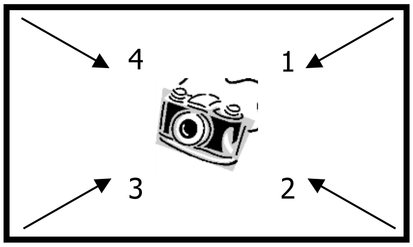


*^ Stand near one corner at a time in each space, face the opposite corner, and take a photo. Repeat for all corners (examples below). The entire room should be captured, and each photo should contain both floor and ceiling.*

*^ If children are present during photography, NO FACES may be photographed.*

***Cafeteria example photos:***

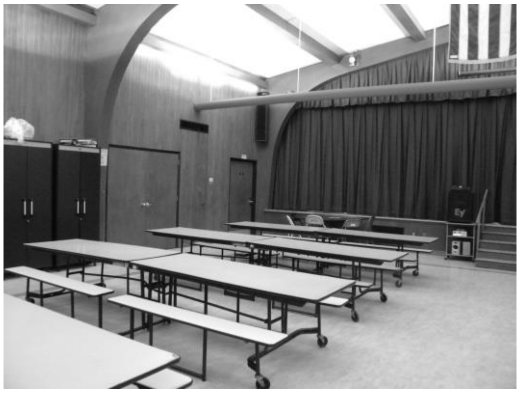

**Corner 1**

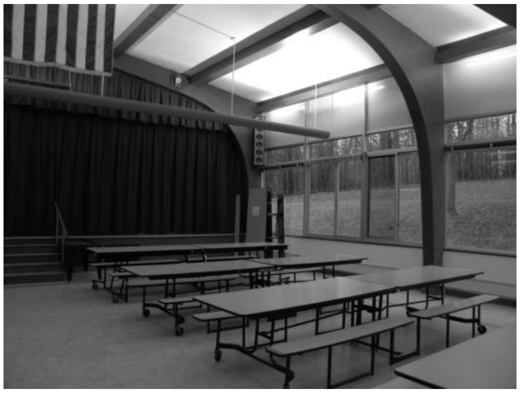

**Corner 2**

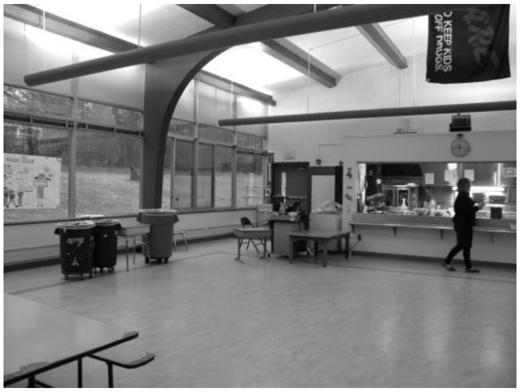

**Corner 3**

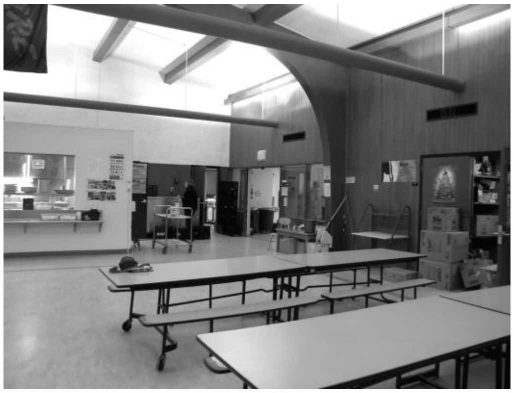

**Corner 4**

***Serving station example photos (be sure to label each item in your sketches on p. 11)***

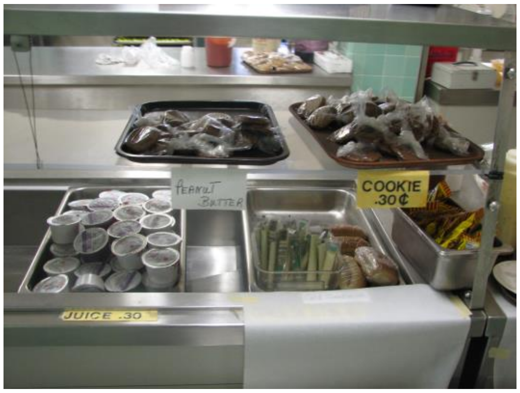


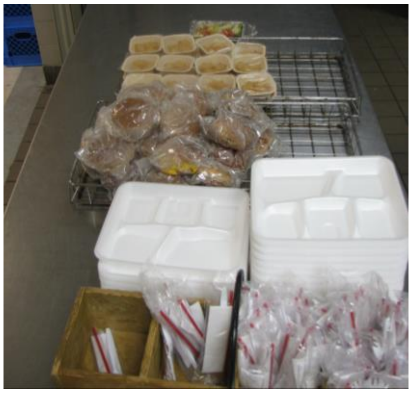

Observer name: ________________________________________ State _______________ School ID#_________

**V. Below, please *sketch and label* the food/beverage preparation (kitchen), serving, and eating areas (cafeteria).**

1. Draw the walls.
2. Draw doors/windows.
3. Draw the furniture.

5. Draw and label:

- ☐ *Kitchen/prep area*
- ☐ *Serving area*
- ☐ *Menu locations* (**M**)
- ☐ *Storage areas*
- ☐ *Student entry/exit*
- ☐ *Number all food/beverage locations* (**#**)
- ☐ *Payment location*
- ☐ *Student circulation paths: entry, seating, food/bev, payment, trash, exit*
- ☐ *Disposal/trash area*
- ☐ *Tables and seating* (draw exact number of tables, note # chairs, bench)
- ☐ *Healthy food and PA signage* (**S**)

**Example sketch:**

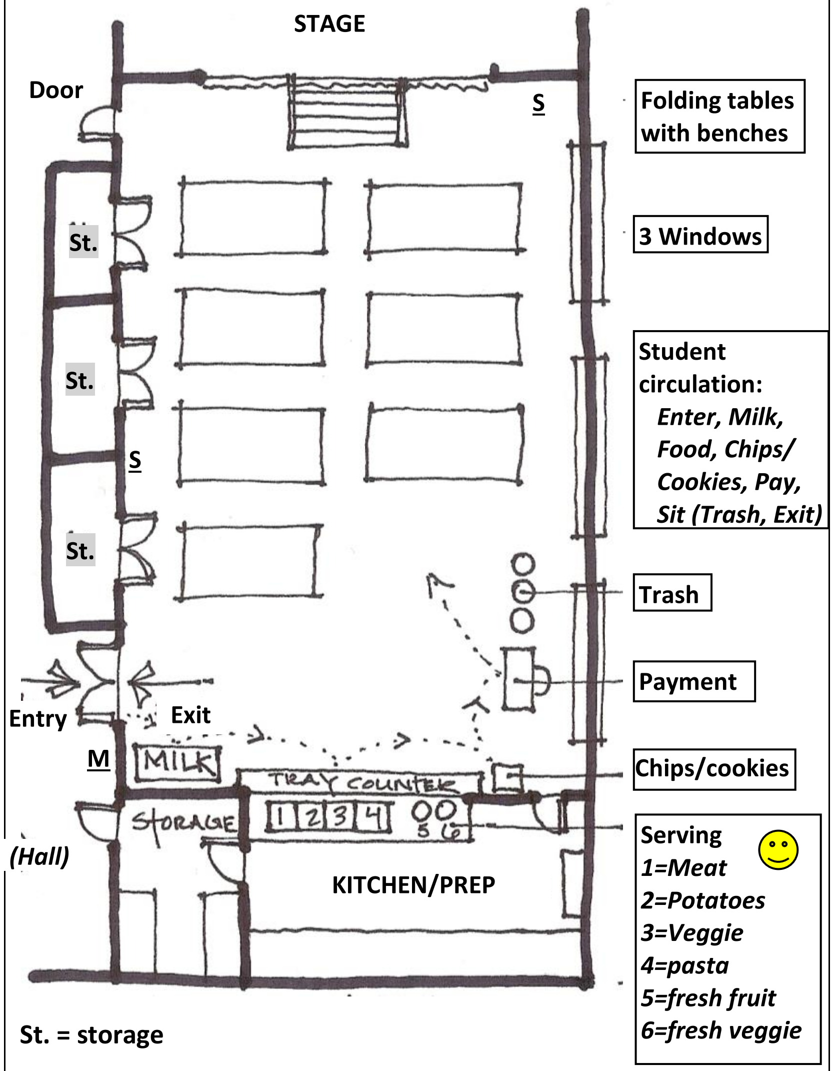

Observer name: ___________________________________ State ______________ School ID#________

W. Below, please photograph, sketch, AND label *each* food and beverage serving location according to the instructions.

**Example sketch: #n/a. MILK refrigerator**

*Front of stainless steel refrigerator*

| | |
|---|---|
| 2% | Chocolate |
| Skim | Strawberry |

*View after student lifts opaque top; beverage label is printed on cardboard cartons; cartons are all the same except for label color/text*

**Example sketch: #1-6. Warm food serving station; fruit bowls**

*Front*

| | | | | |
|---|---|---|---|---|
| Mashed potatoes *(card label)* | Peas Carrots *(card label)* | Chicken patty *(card label)* | Whole apples | Whole bananas |

*Metal trays, uncovered, not behind glass on metal counter top; food items labeled cover descriptively on white card with black handwritten text*

*Opaque red glass bowls on metal labeled counter, no item label*

**INSTRUCTIONS**:

Carefully *photograph* each food and beverage serving location numbered on your previous sketch.

*Sketch* each food and beverage serving location numbered on your previous sketch:

**A. List # from previous sketch**

**B. Title the station**

**C. Draw the station**

**D**. Label individual items

**E. Are individual items labeled?** If yes, describe item labels.

**F**. Describe how each item is served:

***- Container type***
*e.g. tray, bowl, basket, pot*

***- Container material***
*e.g. metal, glass, plastic*

-*Is container* ***transparent or opaque?***

-Is the container ***covered*** *(i.e., students have to remove before serving)?*

-Is the ***cover opaque*** or ***transparent***?

**- Are students served or serve themselves?**

**- Are items pre-portioned?**

**-Describe surfaces below serving containers and materials** *e.g., Metal or glass counter, plastic/metal*

**Observer name**: ________________________________ **State** ______________ **School ID#**________

**Additional Notes** *(contact info, intervention suggestions, relevant policies and programs e.g., recess before lunch, fruit and vegetable snack or backpack program, etc.):*

____________________________________________________________________________________________
____________________________________________________________________________________________
____________________________________________________________________________________________
____________________________________________________________________________________________
____________________________________________________________________________________________
____________________________________________________________________________________________
____________________________________________________________________________________________
____________________________________________________________________________________________
____________________________________________________________________________________________
____________________________________________________________________________________________
____________________________________________________________________________________________
____________________________________________________________________________________________
____________________________________________________________________________________________
____________________________________________________________________________________________
____________________________________________________________________________________________
____________________________________________________________________________________________
____________________________________________________________________________________________
____________________________________________________________________________________________
____________________________________________________________________________________________

## K9 CODING AND SCORING INSTRUCTIONS

----------------------------------------------------------------------------------------------------

*Select the appropriate required "**SF/student**" value(s) from the table below & calculate one of the following formulas. If all cafeteria/eating area table & chair types are the same, use the 1st formula; if not, use the 2nd formula (see **K2.1-4**). You will also need several responses from previous Questions: B3.6, B3.8, I2; K2.1 – K2.4 (2nd formula only).*

**Seating Type**

| **Table type** | ***Attached*** | ***Detached*** |
|---|---|---|
| ***Rectangular*** | 10 SF/student | 11 SF/student |
| ***Round/square*** | 14 SF/student | 15 SF/student |

*This table displays the amount of cafeteria square footage (SF) per student that is required by code, based on the table and chair type.*

**- If all tables & chairs *are the same*:** (**B3.6** # students) / (**B3.8** # meal periods) x (**SF/student**) = **required SF** for scoring

______________ / _________________ x __________ = __________ SF

**- If all tables & chairs are *not the same***: Calculate the **required SF** based on *each* available table/chair type, as applicable.

[(B3.6) / (B3.8) x (**SF/student**) x (**K2.1**) / (K2.1 + K2.2 + K2.3 + K2.4)] + ______________________________ +

[(B3.6) / (B3.8) x (**SF/student**) x (**K2.2**) / (K2.1 + K2.2 + K2.3 + K2.4)] + ______________________________ +

[(B3.6) / (B3.8) x (**SF/student**) x (**K2.3**) / (K2.1 + K2.2 + K2.3 + K2.4)] + ______________________________ +

[(B3.6) / (B3.8) x (**SF/student**) x (**K2.4**) / (K2.1 + K2.2 + K2.3 + K2.4)] ______________________________

**= required SF** = __________ SF

**K9. Cafeteria crowding scoring:** Enter **0 if I2.** (actual area SF) ____________ is *less* than **required SF** calculated above

Enter **1 if I2.** (actual area SF) ____________ is *more* than **required SF** calculated above
